# Evaluating the Associations of Adiposity, Functional Status, and Anthropometric Measures with Nutritional Status in Chronic Hemodialysis Patients: A Cross-Sectional Study

**DOI:** 10.3390/nu17193034

**Published:** 2025-09-23

**Authors:** Martyna Andreew-Gamza, Beata Hornik

**Affiliations:** 1 Doctoral School, Medical University of Silesia in Katowice, 40-055 Katowice, Poland; 2 Department of Internal Nursing, Faculty of Health Sciences in Katowice, Medical University of Silesia in Katowice, 40-635 Katowice, Poland

**Keywords:** nutritional assessment, adipose tissue, bioelectrical impedance analysis, VAI, BAI, hemodialysis, phase angle, handgrip strength

## Abstract

Background: Malnutrition is common in chronic hemodialysis (HD) patients and often remains underdiagnosed. While body composition, functional status, and anthropometric measures can support nutritional assessment, their associations with nutritional status are not fully established in this population. This study aimed to evaluate the diagnostic performance of various measures for assessing malnutrition in chronic HD patients, using the Subjective Global Assessment (SGA) as the reference standard. Methods: This cross-sectional study involved chronic HD patients, stratified by nutritional status using the SGA. Data collection consisted of clinical interviews, anthropometric and functional measurements, bioelectrical impedance analysis (BIA), and biochemical analyses. Statistical analysis included Spearman’s correlation, logistic regression, receiver operating characteristic (ROC) curve analysis with area under the curve (AUC) to assess predictive accuracy, standardized effect sizes to show the magnitude of differences, and kappa statistics to evaluate concordance between variables. Results: This study included 103 chronic HD patients. Malnutrition was diagnosed in 50.5% of patients based on the SGA. Phase angle (PA) was the strongest single predictor of malnutrition (AUC = 0.79; specificity 0.88; sensitivity 0.58). PA ≤ 5.1° was significantly associated with higher malnutrition risk (OR: 10.23; 95% CI: 3.93–30.61; *p* < 0.001). Handgrip strength (HGS) also demonstrated good diagnostic value (AUC = 0.71; specificity 0.84; sensitivity 0.59). A multivariable model incorporating eight parameters—gender, post-dialysis ECW/ICW ratio, post-dialysis lean and fat mass, serum albumin, normalized protein catabolic rate (nPCR), arm circumference (AC), and HGS—achieved an AUC of 0.88 (95% CI: 0.81–0.95) and pseudo-R^2^ of 0.46, demonstrating improved predictive performance. Conclusions: An integrated panel of anthropometric, bioimpedance, functional, and biochemical markers provides superior diagnostic accuracy compared to individual predictors, supporting a holistic diagnostic approach in HD patients.

## 1. Introduction

Many patients undergoing chronic hemodialysis (HD) are at risk of protein–energy malnutrition (PEM), with prevalence estimates ranging from 20% to 60% [1]. This condition is multifactorial, resulting from chronic inflammation, metabolic acidosis, nutrient losses during dialysis, reduced appetite, and inadequate intake. Protein–energy wasting (PEW), a related but broader syndrome, involves both nutritional and metabolic disturbances and is characterized by loss of muscle and fat mass [2,3,4,5,6,7,8]. The International Society of Renal Nutrition and Metabolism (ISRNM) defined diagnostic criteria for PEW across four categories: biochemical markers, body mass, muscle mass, and dietary intake. A diagnosis requires meeting at least one criterion in three of the four categories [9].

According to the European Best Practice Guidelines, nutritional status in HD patients should be assessed at the start of renal replacement therapy and, subsequently, at regular intervals depending on age and dialysis duration [4]. The most frequently recommended tools include the Subjective Global Assessment (SGA), the Malnutrition-Inflammation Score (MIS), and the Mini Nutritional Assessment (MNA) [2,3,4,5,6,7,8], with the validated SGA being the most widely used and recommended due to its comprehensive clinical approach [2]. The SGA has demonstrated strong diagnostic value, integrating dietary intake, clinical symptoms, and physical findings [10]. Additionally, the Geriatric Nutritional Risk Index (GNRI) and diagnostic criteria proposed by the Global Leadership Initiative on Malnutrition (GLIM) are also utilized in nutritional assessment of HD patients, but they differ in the diagnostic approach and scope of evaluated parameters [11,12].

Nutritional status significantly influences body composition and adipose tissue distribution. In HD patients, commonly assessed markers include waist-to-hip ratio (WHR), body fat mass, and bioelectrical impedance analysis (BIA)-derived indicators such as lean body mass, muscle mass, and fluid status [2]. Although visceral adiposity index (VAI) and body adiposity index (BAI) may provide additional insights, their clinical utility in this population remains limited [13]. Notably, indices such as body mass index (BMI) and WHR are more predictive of the cardiometabolic risk than of malnutrition, particularly as they are unable to reflect reductions in lean body mass in obese individuals [14].

Simple anthropometric measurements such as calf circumference (CC) and arm circumference (AC) are practical, noninvasive indicators of muscle mass and are associated with outcomes such as mortality and prolonged hospitalization [12,15]. Similarly, handgrip strength (HGS) is a reproducible functional marker that reflects muscle function and is increasingly used as part of nutritional assessment, supported by SGA and by recent National Kidney Foundation Kidney Disease Outcomes Quality Initiative (K/DOQI) guidelines [2,15]. Phase angle (PA), a BIA-derived parameter designed primarily for body composition, also shows potential as a marker of malnutrition and inflammation by reflecting cellular integrity, though its interpretation in dialysis patients can be confounded by fluid status [16,17,18]. HGS and simple anthropometric measurements are time-efficient and can be performed by all members of the interdisciplinary team. Although more time-consuming, BIA is increasingly implemented in dialysis units, primarily as a tool supporting fluid status assessment. Additionally, BIA provides clinically relevant parameters that may be valuable in nutritional assessment, such as adiposity and cellular integrity as reflected by PA.

While anthropometric, bioimpedance, and functional parameters cannot individually replace a comprehensive nutritional assessment, examining their interrelationships is clinically relevant. These parameters may provide reliable and objective measures for early identification of malnutrition risk and for longitudinal monitoring of nutritional status. Such early detection is essential for implementing timely and effective preventive or therapeutic interventions, particularly in high-risk populations such as HD patients.

Our approach does not challenge the fundamental role of the SGA as a practical, evidence-based method for nutritional assessment [2,3,4,5,6,7,8]. Despite extensive research on nutritional assessment in HD patients, few studies have simultaneously evaluated a broad spectrum of anthropometric, bioimpedance, and functional parameters in relation to the SGA. We assumed that adiposity, functional status, and anthropometric measures are associated with nutritional status in HD patients. We hypothesized that, in addition to the SGA, other measures—used individually or in combination—can accurately identify malnutrition in this population.

The aim of this study was to evaluate associations and the diagnostic performance of different measures for identifying malnutrition in chronic HD patients, compared to the SGA as the reference standard, and to determine the potential combination of parameters for the accurate differentiation of malnutrition from normal nutritional status.

## 2. Materials and Methods

In this cross-sectional observational study, data collection included clinical interviews, anthropometric measurements, bioimpedance measurements, functional measurements, and biochemical analyses. The manuscript was prepared according to the Strengthening the Reporting of Observational Studies in Epidemiology (STROBE) guidelines for observational studies [19].

### 2.1. Study Population

**Figure 1:**
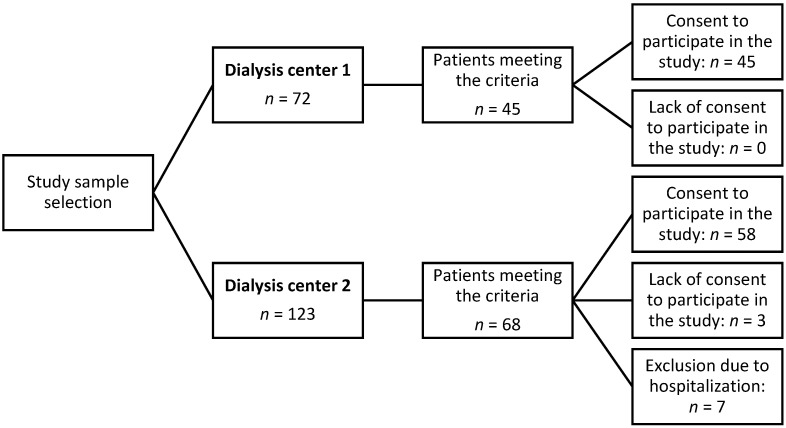
Study sample selection process.

This cross-sectional observational study involved chronic HD patients. It was conducted at two dialysis centers located in Southern Poland. This study was carried out between 2021 and 2024. All patients who met the inclusion and exclusion criteria and provided informed consent were enrolled in the study (Figure 1).

Inclusion criteria involved age over 18 years, stage 5 chronic kidney disease (CKD), undergoing HD for at least 3 months, informed and voluntary consent to participate in the study, and a health condition allowing for coherent verbal communication.

Exclusion criteria included the exacerbation of clinical condition, dyspnea classified as New York Heart Association (NYHA) class III/IV, malignancy, liver cirrhosis, chronic infectious diseases, diseases accompanied by high fever, malabsorption syndrome, inability to maintain a stable standing posture, confusion/advanced dementia, aphasia, and a lack of consent to participate in the study. Additional exclusion criteria were included in the Supplementary Material (Supplemental Methods Section).

The study sample size was calculated using Cochran’s sample size formula for a known population size [20]. As a result, the representative sample size for this study was 88 at a 95% confidence level, assuming a 50% population proportion and an acceptable sampling error of 5%.

All procedures carried out during the study were in accordance with ethical standards consistent with the Declaration of Helsinki of 1964, as well as with the standards adopted by the Medical University of Silesia in Katowice and the dialysis centers where this study was conducted. Each patient received an information sheet detailing the course of the planned study and the principles of personal data processing, in accordance with Regulation (EU) 2016/679 of the European Parliament and of the Council of 27 April 2016 on the protection of natural persons with regard to the processing of personal data and on the free movement of such data, repealing Directive 95/46/EC (General Data Protection Regulation).

Each participant provided informed consent to take part in this study and was informed of their right to withdraw consent at any time during this study. The study was approved by the Bioethics Committee of the Medical University of Silesia in Katowice (Resolution No. PCN/CBN/0022/KB1/98/21 concerning the opinion on the medical research project, dated 21 September 2021).

### 2.2. Measurements

The dialysis adequacy index (*Kt*/*V*) was calculated using Daugirdas’ equation, which involves the natural logarithm of the ratio of pre- to post-dialysis urea concentrations [21]. For the purposes of this study, the values were obtained from the patients’ medical records.

Additionally, the urea reduction ratio (URR) was calculated based on the following formula:

$$URR=\left(\frac{C_{0}-C_{t}}{C_{0}}\right)\times 100$$

where *C*_0_ is the urea concentration before the hemodialysis session and *C_t_* is the urea concentration after the hemodialysis session [22]. In the studied patients, the ultrafiltration volume (UFV)—calculated as the difference between total body weight before and after dialysis—was determined.

### 2.3. Survey Questionnaires

#### 2.3.1. Self-Designed Survey Questionnaire

A structured interview was conducted, consisting of 15 questions related to sex, age, the education level, place of residence, marital status, social status, type of occupation, type of vascular access, time of initiation of renal replacement therapy, residual diuresis volume, history of hospitalization in the year preceding the study and its causes, and underlying cause of CKD.

#### 2.3.2. Subjective Global Assessment

K/DOQI and the European Best Practice Guidelines on Nutrition (EBPG) guidelines recommend the SGA as a reliable and essential method for evaluating the nutritional status of individuals with kidney disease [2,4]. It enables a screening assessment of nutritional status and the determination of its severity. Despite the subjectivity of this method (which depends on the evaluator’s experience in assessing the patient’s condition), it is a frequently used and reliable tool [6,23].

In the present study, the 7-point version of the SGA was used. The SGA consisted of a summary of the interview—with both objective and subjective components (questions regarding age, height, sex, body weight, type of food consumed, changes in dietary habits, and physical performance)—as well as findings from the physical examination (reduction in subcutaneous fat over the triceps muscle and chest, atrophy of the quadriceps and deltoid muscles, sacral edema, ankle edema, and ascites). Normal or well-nourished status (equivalent to global SGA category A) was defined as a score of 6–7 on the 7-point SGA scale. Malnutrition was defined as a score of ≤5 [3,24] on the 7-point SGA scale, where scores 3–5 indicated mild-to-moderate malnutrition (equivalent to global SGA category B) and scores 1–2 indicated severe malnutrition (equivalent to global SGA category C). Nutritional status assessment using the SGA scale should be performed every six months [2,15,23].

### 2.4. Anthropometric and Functional Measurements

#### 2.4.1. Handgrip Strength Measured Using Dynamometer

HGS [kg] was used as an indicator of functional status. Measurements were performed using a handheld dynamometer with an accuracy of 100 g and a maximum measurement range of up to 80 kg. HGS was measured prior to the HD session. For patients with an arteriovenous fistula, the measurement was performed on the hand of the upper limb without the fistula; for those with a central venous catheter, the dominant hand was used. During the measurement, the patient remained in a standing upright position with arms relaxed and hanging freely. The patient performed two handgrip trials with a 15 s interval, and the best result was recorded. The procedure followed the methodology applied in studies by other authors [24,25,26,27].

The HGS test is a proven and recommended method for assessing the nutritional status and muscle strength of HD patients [28]. The European Working Group on Sarcopenia in Older People (EWGSOP) recommends evaluating muscle strength based on the patient’s sex and BMI, defining low muscle strength as follows: men: BMI ≤ 24: ≤29 kg, BMI 24.1–28: ≤30 kg, BMI > 28: ≤32 kg; women: BMI ≤ 23: ≤17 kg, BMI 23.1–26: ≤17.3 kg, BMI 26.1–29: ≤18 kg, BMI > 29: ≤21 kg [29].

#### 2.4.2. Arm Circumference and Calf Circumference

Additional methods used in this study included the measurements of AC [cm] and CC [cm]. The cutoff points were set at an AC value ≤ 22 cm and a CC value ≤ 31 cm [30]. These methods are applicable in the evaluation of the nutritional status of HD patients [31].

The measurements of AC and CC were performed twice on the limb without the arteriovenous fistula using a non-stretch measuring tape, and the mean value was calculated. The mid-upper AC measurement was performed with the patient’s upper arm relaxed alongside the body, at the midpoint of the line from olecranon to shoulder peak of the upper arm. The CC was measured at the thickest part of the gastrocnemius muscle of the lower leg [31].

#### 2.4.3. Body Mass Index

The BMI [kg/m^2^] was calculated using the following formula: body weight [kg]/height [m]^2^. The cutoff point was set at a value < 23 kg/m^2^ [32].

#### 2.4.4. Waist-to-Hip Ratio

To determine body fat distribution, the WHR was calculated using the following formula: waist circumference [cm]/hip circumference [cm]. WHR values < 0.8 in women and <1.0 in men indicate visceral fat tissue within normal range. Visceral obesity is defined as a WHR ≥ 0.8 in women and ≥1.0 in men. This index is widely used in studies involving HD patients [33].

### 2.5. Body Composition Measurements

Body composition was analyzed using the InBody S10 Body Composition Analyzer (InBody Co., Ltd., Seoul, Republic of Korea). This noninvasive examination is based on the method of BIA. This method enabled the highly accurate estimation of, among other parameters, total fat mass and lean body mass, which was of significant importance for body composition assessment. The measurement technique relies on multifrequency bioimpedance analysis using an 8-point tactile electrode system. Segmental measurement was performed (right and left upper limb, lower limb, and trunk). The examination was conducted both before and after the HD session; in the present study, the results obtained after the HD session were included.

The analysis included the following parameters: post-dialysis fat tissue content (%); mass of body fat (MBF) [kg]; and PA [°]—a phase angle determined based on resistance and reactance values obtained through BIA, calculated according to the following formula:

$$PA=arctan\left(\frac{X_{c}}{R}\right)\times \frac{180{}^{\circ}}{\pi }$$

where *X_c_* is the reactance [Ω]—associated with the capacitance of cell membranes; *R* is the resistance [Ω] (tissue resistivity)—primarily associated with water content; and arctan is a trigonometric function (arcus tangent), which converts the ratio *X_c_*/*R* into an angle [18]. The cutoff points were set at a fat tissue content value < 10% [11] and a PA value ≤ 5° [16,17,34].

The following parameters were included in the analysis: skeletal muscle mass (SMM) [kg], lean body mass after dialysis, extracellular water (ECW) [L], intracellular water (ICW) [L], ECW index, and the extracellular to intracellular water (ECW/ICW) ratio. Other authors have confirmed the significance of BIA in assessing nutritional status and diagnosing PEW in HD patients [35,36].

### 2.6. Laboratory Tests and Adipose Tissue Distribution Indices

#### 2.6.1. Laboratory Tests

Blood samples were collected in a fasting state prior to the HD session—at least 12 h after the last meal for individuals participating in the morning dialysis session, or after a 4 h fast for patients scheduled for afternoon or evening dialysis.

Laboratory tests included the measurement of albumin concentration, as well as total protein, transferrin, total cholesterol, high-density lipoprotein (HDL) [mmol/L], low-density lipoprotein (LDL) [mmol/L], triglycerides (TG) [mmol/L], high-sensitivity *C*-reactive protein (hs-CRP) [mg/L], post-dialysis urea [mmol/L], creatinine [mg/dL], and normalized protein catabolic rate (nPCR) [g/kg/day] [37].

#### 2.6.2. Visceral Adiposity Index and Body Adiposity Index

In the present study, the VAI was used to assess visceral adipose tissue dysfunction and cardiometabolic risk, as well as the BAI, which provides information on the percentage of body fat without the need for weighing. The formulas for calculating the VAI differ depending on sex. To determine the VAI, the following parameters are required: BMI, waist circumference, HDL concentration, and TG concentration [38].

The VAI for the male participants in this study was calculated using the following formula:

$$VAI=\left(\frac{WC}{39.68+\left(1.88\;\times \;BMI\right)}\right)\times \;\left(\frac{TG\left[\frac{mmol}{L}\right]}{1.03}\right)\times \;\left(\frac{1.31}{HDL\left[\frac{mmol}{L}\right]}\right)$$

and for female participants, the VAI was calculated using the following formula:

$$VAI=\left(\frac{WC}{36.58+\left(1.89\;\times \;BMI\right)}\right)\times \;\left(\frac{TG\left[\frac{mmol}{L}\right]}{0.81}\right)\times \;\left(\frac{1.52}{HDL\left[\frac{mmol}{L}\right]}\right)$$

To determine the BAI, hip circumference and height are required. The BAI was calculated using the formula below, regardless of sex [39]:

$$BAI=\frac{hip\;circumference\;[cm]}{{\left(height\;[m]\right)}^{1.5}}-18$$

### 2.7. Outcome Variables

The following variables were defined in the study as primary outcome variables: SGA, HGS, bioimpedance parameters (post-dialysis PA [°], post-dialysis SMM [kg], post-dialysis lean body mass [kg], post-dialysis MBF [kg], post-dialysis fat tissue content), BMI [kg/m^2^], AC [cm], CC [cm], serum albumin [g/L], and nPCR [g/kg/day].

The following variables were defined as secondary outcome variables: other bioimpedance parameters (post-dialysis body weight [kg], post-dialysis ECW index, post-dialysis ECW [L], post-dialysis ICW [L], post-dialysis ECW/ICW ratio), laboratory tests (TG [mmol/L], HDL [mmol/L], LDL [mmol/L]), WHR, BAI, and VAI.

### 2.8. Statistical Analysis

Statistical analysis was conducted using the software Statistica 13.1 (StatSoft, Inc., Tulsa, OK, USA). This study included the evaluation of both quantitative and qualitative variables. To characterize the structure of the examined variables, basic descriptive statistics were calculated, including measures of central tendency and variability. Additionally, 95% confidence intervals (CI) for the mean values were computed. The Shapiro–Wilk test was used to verify the normality of distributions. Results were presented as mean and standard deviation (M ± SD) and as median (Me) and interquartile range (IQR) boundaries. The Student’s *t*-test for independent samples was applied to assess the significance of differences for parametric variables, while the Mann–Whitney *U* test was used for non-parametric variables. Relationships between variables were assessed using Spearman’s rank correlation coefficient (*ρ*) and—for ordinal variables—Kendall’s tau-b coefficient (*τ*_b_), a non-parametric measure of association. For variables measured on ordinal and nominal scales, the chi-square test (χ^2^) was applied, comparing observed frequencies with expected frequencies under the null hypothesis (of no association between the two variables).

Standardized effect sizes (Cohen’s *d*) were used to quantify the magnitude of differences between measurements stratified by the cutoff points. Inter-rater agreement between ordinal and nominal variables stratified by the cutoff points and categorical variable consistency analyses were conducted using kappa statistics (Cohen’s κ), which accounts for agreement occurring by chance.

Logistic regression models were also constructed, and receiver operating characteristic (ROC) curves were generated, allowing objective determination of thresholds maximizing the balance between sensitivity and specificity. Optimal cutpoints were estimated for 14 individual clinical parameters to differentiate malnutrition (including cachexia) from normal nutritional status, as defined by the SGA. Thresholds were determined with SGA as the classification variable, designating “malnutrition or cachexia” as the positive class. The maximize metric method was employed, optimizing Youden’s index (defined as sensitivity + specificity − 1) to balance diagnostic performance. In instances where multiple optimal cutpoints were identified, tie-breaking was applied to select the most clinically relevant threshold. Bootstrap resampling with 2000 iterations was performed to assess the stability of the thresholds and associated metrics. Diagnostic metrics, including accuracy, sensitivity, specificity, Youden’s index, and prevalence (the proportion of the positive class in the dataset), were calculated at each optimal threshold. The area under the receiver operating characteristic curve (AUC) was computed to evaluate discriminatory ability, with 95% confidence intervals derived using bootstrap methods. A significance level of *p* < 0.05 was adopted for all analyses.

To construct a multivariable model—incorporating a panel integrating multiple independent complementary parameters associated with malnutrition—as an alternative to the univariate approach, logistic regression analysis was performed, aiming to enhance diagnostic reliability and discriminatory power. A stepwise procedure employing backward elimination was utilized for variable selection, guided by the Akaike information criterion (AIC) to identify the most parsimonious model. Multicollinearity among the selected predictors was assessed using variance inflation factors (VIFs), with a threshold of VIF < 5 indicating acceptable independence. Model performance was evaluated using pseudo-R^2^, and odds ratios (ORs) with 95% CI were reported for each predictor. Statistical significance was defined at an alpha level of 0.05, with *p*-values < 0.05 considered significant and those between 0.05 and 0.10 noted as borderline significant. The discriminatory abilities of the univariate and multivariable models were compared using ROC curves. The DeLong test was applied to assess the statistical significance of differences in AUC values between the two competitive models.

## 3. Results

### 3.1. Baseline Characteristics of the Patients (Table 1)

The study included 103 HD patients with a mean age of 61 ± 14 years (M ± SD), ranging from 28 to 90 years. The cohort consisted of 39 women (37.9%) and 64 men (62.1%). According to the SGA classification, 51 participants (49.5%) were normal or well-nourished (SGA-A), 45 individuals (43.7%) were mildly to moderately malnourished (SGA-B), and severe malnutrition (SGA-C) was found in seven patients (6.8%). Given the small sample size in SGA-C, the SGA-B and SGA-C categories were combined for analysis. The demographic and clinical characteristics of the studied patients are presented in Table 1 summarizing parameter differences between the SGA-(B + C) and SGA-A subgroups.

SGA-(B + C) patients were significantly older than SGA-A patients (64 ± 14 vs. 58 ± 15 years; *p* = 0.044). HC was significantly lower in SGA-(B + C) patients compared to SGA-A patients (95.6 ± 13.1 cm vs. 99.9 ± 10.3 cm; *p* = 0.013, *d* = −0.37). Similarly, AC was reduced in SGA-(B + C) patients compared to SGA-A patients (27.4 ± 4.2 cm vs. 30.5 ± 4.1 cm; *p* = 0.001, *d* = −0.74). CC was also significantly smaller in SGA-(B + C) patients compared to SGA-A patients (34.8 ± 4.6 cm vs. 36.9 ± 4.2 cm; *p* = 0.003, *d* = −0.48). The effect sizes (Cohen’s *d*) indicated moderate differences between the SGA-(B + C) and SGA-A patients.

Significantly lower HGS values were observed in SGA-(B + C) individuals compared to those SGA-A (25.1 ± 13.1 kg vs. 32.3 ± 9.4 kg; *p* < 0.001), with moderate difference between groups (*d* = −0.63). Hydration markers also showed significant differences: ECW (*p* = 0.039), ICW (*p* < 0.001), and their ratio (ECW/ICW) (*p* < 0.001), as well as PA, which was lower in SGA-(B + C) patients (*p* < 0.001, *d* = −1.13). Creatinine and albumin concentrations were significantly lower in these patients as well (*p* < 0.001 and *p* = 0.001, respectively). The effect sizes indicated moderate-to-large differences between the SGA-(B + C) and SGA-A patients.

### 3.2. Assessment of Nutritional Status and Adiposity in the Studied Group (Table 2)

Table 2 presents the numbers and percentages of patients falling below and above the established cutoff values for selected parameters, along with the results of χ^2^ with Yates’ correction, Kendall’s *τ*_b_, and Cohen’s κ statistics. Significant associations were observed for HGS (*p* = 0.041), indicating a higher prevalence of reduced HGS in SGA-(B + C) patients, with a weak agreement with SGA (κ = 0.21). A significantly higher proportion of patients with AC ≤ 22 cm was found in the SGA-(B + C) subgroup (*p* = 0.011), with weak-to-moderate association and limited agreement with SGA (*τ*_b_ = 0.29; κ = 0.153). Similarly, CC ≤ 31 cm was more prevalent among SGA-(B + C) patients (83.3% vs. 16.7%; *p* = 0.035). A significantly higher proportion of patients with post-dialysis PA ≤ 5° was classified as SGA-(B + C) compared to SGA-A (81.8% vs. 18.2%; *p* < 0.001), indicating moderate association and fair-to-moderate agreement with SGA (*τ*_b_ = 0.43; κ = 0.400).

### 3.3. Determination of Relationship Between Nutritional Status Assessed Using Subjective Global Assessment and Indicators of Fat Tissue Distribution and Body Adiposity (Body Adiposity Index, Visceral Adiposity Index), as Well as Selected Parameters Obtained Through Bioelectrical Impedance Analysis in Groups of Studied Hemodialysis Patients (Table 3)

In subsequent analyses, basic descriptive statistics were presented for indicators of fat tissue distribution and body adiposity (BAI and VAI), as well as selected body composition analysis parameters, in relation to the results of the SGA questionnaire evaluating the overall nutritional status of the participants (Table 3).

The analysis of the results presented in Table 3 revealed statistically significant negative correlations between the nutritional status levels—ranging from normal status to cachexia according to the SGA—and several variables, including post-dialysis body weight [kg] (*ρ* = −0.38; *p* < 0.001), post-dialysis SMM [kg] (*ρ* = −0.39; *p* < 0.001), post-dialysis lean body mass [kg] (*ρ* = −0.32; *p* = 0.001), and post-dialysis PA [°] (*ρ* = −0.46; *p* < 0.001). It was found that, as the values of these variables increased, the degree of malnutrition assessed using the SGA decreased. The analysis also identified two statistically significant weak positive correlations between the SGA and the following variables: post-dialysis ECW index (*ρ* = 0.39; *p* < 0.001), and ECW/ICW ratio (*ρ* = 0.39; *p* < 0.001). It was found that, as the values of these variables increased, the degree of malnutrition, as assessed by the SGA, also increased.

### 3.4. Diagnostic Performance of Clinical Parameters and Identification of Optimal Thresholds for Differentiating Malnutrition from Normal Nutritional Status (Table 4)

The optimal thresholds for a comprehensive set of clinical parameters, as presented in Table 4, facilitated the differentiation of malnutrition (including cachexia) from normal nutritional status, utilizing the SGA as the reference standard.

Parameters with predictive potential, characterized by an AUC with a 95% CI lower bound exceeding 0.50, included post-dialysis ECW/ICW ratio (AUC = 0.77; 95% CI: 0.63–0.88), post-dialysis PA (AUC = 0.79; 95% CI: 0.66–0.89), post-dialysis lean body mass (AUC = 0.66; 95% CI: 0.51–0.80), serum albumin (AUC = 0.69; 95% CI: 0.53–0.82), AC (AUC = 0.68; 95% CI: 0.53–0.82), CC (AUC = 0.68; 95% CI: 0.52–0.81), HGS (AUC = 0.71; 95% CI: 0.58–0.85), and gender (AUC = 0.60; 95% CI: 0.51–0.70). These metrics demonstrated moderate-to-good discriminatory ability, reflecting their relevance in identifying nutritional deficits in dialysis patients. Conversely, some parameters included in our assessment demonstrated limited predictive value, with lower CI bounds at or below 0.50, indicating they may not reliably distinguish malnutrition in isolation and, therefore, show limited diagnostic utility. Although nPCR had high sensitivity, its low specificity and an AUC close to random chance limit its diagnostic usefulness and may result in a high rate of false positives (Table 4).

Among the parameters with predictive potential, PA emerged as the best single predictor demonstrating good classification ability and overall diagnostic effectiveness, based on its highest AUC (0.79) and Youden’s index (0.46), alongside robust specificity (0.88) and moderate sensitivity (0.58). The cutpoint for PA was determined at 5.1° based on ROC curve analysis. HGS followed with an AUC of 0.71 and a Youden’s index of 0.43, offering balanced sensitivity (0.59) and specificity (0.84). Serum albumin (AUC = 0.69) and ECW/ICW ratio (AUC = 0.77) also demonstrated solid discriminatory performance, with the latter highlighting fluid imbalances that often accompany cachexia. Serum albumin, despite high specificity, demonstrated low sensitivity and only moderate predictive value. Anthropometric measures, i.e., AC (AUC = 0.68; sensitivity 0.35; specificity 0.94) and CC (AUC = 0.68; sensitivity 0.87; specificity 0.43), provided complementary insights into muscle wasting, though their imbalances in sensitivity and specificity limit standalone application. Gender, as a categorical variable (with female coded as the positive class), demonstrated an AUC of 0.60, indicating modest discriminatory performance, potentially linked to gender-specific physiological vulnerabilities in malnutrition risk. The accuracy of individual parameters with predictive potential ranged from 0.60 to 0.73. Individual parameters generally displayed limitations, such as moderate sensitivity across the board, which may result in the underdetection of malnutrition cases and variable accuracy (Table 4).

### 3.5. Comparative Logistic Regression Analysis: Multivariable Panel Model Versus Univariate Phase Angle Model for Predicting Malnutrition (Table 5)

To enhance diagnostic robustness, a multivariable panel was constructed to integrate parameters offering complementary physiological and sociodemographic perspectives. Commencing with the initial 15 predictors detailed in Table 4, categorized by their optimal cutpoints, a stepwise procedure employing backward elimination, guided by AIC, culminated in the selection of eight final predictors: gender, post-dialysis ECW/ICW ratio, post-dialysis lean body mass, post-dialysis MBF, serum albumin, nPCR, AC, and HGS (Table 5). Subsequent model evaluation confirmed the absence of multicollinearity, as evidenced by all VIFs remaining below 5, thereby affirming the independence and reliability of the retained variables in the predictive framework.

In the univariate logistic regression model, incorporating solely the best individual predictor, i.e., PA, lower PA values were significantly associated with increased odds of malnutrition, increasing these odds 10-fold for PA ≤ 5.1° compared to > 5.1° (OR: 10.23; 95% CI: 3.93–30.61; *p* < 0.001). This indicates that patients exhibiting a PA at or below this threshold were over 10 times more likely to be classified as malnourished, underscoring its robust standalone predictive value. The model’s pseudo-R^2^ of 0.232 reflected moderate-to-good explanatory power, based on 103 observations.

In contrast, the multivariable model yielding a substantially improved pseudo-R^2^ of 0.460 across 103 observations. This substantial enhancement in model fit when simultaneously considering eight parameters suggests that the integrated panel captured a greater proportion of variance in malnutrition risk compared to PA alone. Within this model, several predictors emerged as statistically significant (*p* < 0.05), providing reliable indicators with direct clinical relevance.

Among the analyzed parameters, HGS ≤ 23.3 kg (OR: 7.54; 95% CI: 1.50–37.90; *p* = 0.011) demonstrated significant predictive strength, indicating that individuals with reduced grip strength were over seven times more likely to exhibit malnutrition. Post-dialysis lean body mass ≤ 39.8 kg (OR: 6.65; 95% CI: 1.20–36.89; *p* = 0.029) further emerged as a reliable predictor, highlighting the critical role of muscle mass depletion in malnutrition pathophysiology.

Borderline significant predictors included male gender (OR: 5.48; 95% CI: 0.90–33.48; *p* = 0.061), indicating a potential heightened vulnerability in males, and AC ≤ 25.5 cm (OR: 5.18; 95% CI: 0.87–30.89; *p* = 0.064), which corroborates anthropometric evidence of upper limb muscle wasting. Other parameters, such as ECW/ICW ratio ≥ 0.66 (OR: 2.41; *p* = 0.142), post-dialysis MBF ≤ 35.3 kg (OR: 2.93; *p* = 0.099), serum albumin ≤ 37.3 g/L (OR: 2.49; *p* = 0.108), and nPCR ≤ 1.19 g/kg/day (OR: 2.47; *p* = 0.173), exhibited elevated ORs but lacked statistical significance, potentially due to sample size limitations; nonetheless, their trends reinforce the multifaceted nature of malnutrition, encompassing fluid imbalances, adipose tissue loss, hypoalbuminemia from inflammation or liver dysfunction, and suboptimal protein utilization (Table 5).

**Figure 2:**
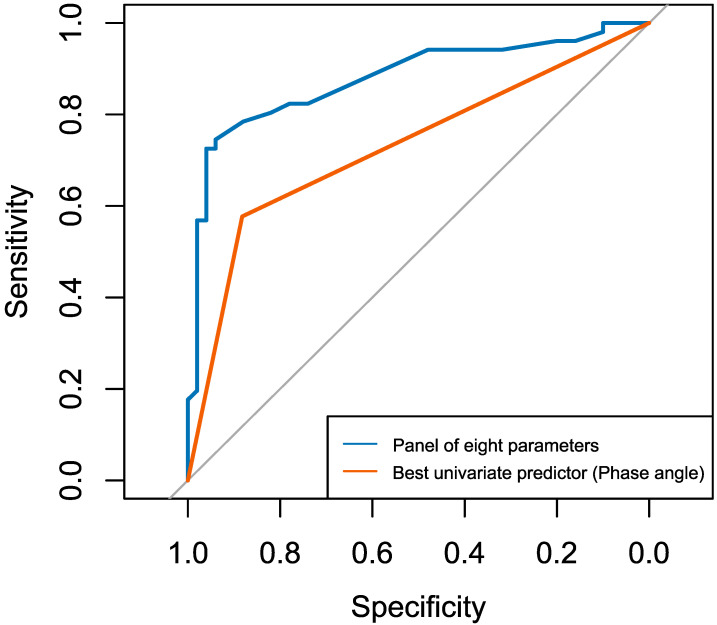
Comparison of receiver operating characteristic (ROC) curves: panel of eight parameters versus best univariate predictor (PA).

The AUC for the multivariable model incorporating a panel of eight parameters, in distinguishing malnutrition (including cachexia) from normal nutritional status, was 0.88 (95% CI: 0.81–0.95) (Figure 2). In comparison, the AUC for the best univariate predictor—PA—was 0.73 (95% CI: 0.66–0.89). DeLong’s test for comparing the two ROC curves revealed a statistically significant difference (D = 2.74; *df* = 198.42; *p* = 0.007), confirming that the difference in AUC values was not attributable to chance under the alternative hypothesis that the true difference is not equal to zero.

### 3.6. Associations Between Functional, Anthropometric, and Bioelectrical Impedance Analysis-Derived Measures (Tables S1–S4)

Detailed results of supplementary analyses, conducted to enhance the interpretation of the primary findings, are provided in the Supplementary Tables S1–S4. In subsequent analyses, the interrelationships among selected measures were evaluated according to predefined cutoff points applied to identify patients with low values of nutritional markers. These cutoff points, as detailed in the Materials and Methods section, were established based on clinical guidelines [11,16,17,29,30,32,33,34]. The associations, along with their corresponding effect sizes, between selected functional (i.e., HGS) and anthropometric (i.e., BMI, AC, CC) measures in relation to indicators of fat tissue distribution and body adiposity (i.e., BAI, VAI), as well as to selected BIA-derived body composition parameters (i.e., fat mass, muscle mass, PA, the ECW/ICW ratio), are presented in Supplementary Table S1.

The logistic regression model for BMI < 23 kg/m^2^, based on BAI and VAI, is summarized in Supplementary Table S2, and the corresponding ROC analysis with optimal cutpoints is presented in Supplementary Figure S1. Agreement between SGA and PA and agreement across multimethod adiposity assessment are presented in Supplementary Table S3. Additionally, stratification by gender of patients participating in the study for the studied sociodemographic and clinical characteristics was performed, with results presented in Supplementary Table S4.

## 4. Discussion

This study demonstrated associations between selected adiposity, functional status, and anthropometric measures with nutritional status in chronic HD patients, providing new data regarding these relationships. Among the individual measures analyzed, PA boasted the greatest ability to distinguish malnourished from well-nourished individuals, standing out as the premier univariate predictor and confirming its value as an objective, complementary parameter. HGS followed closely, demonstrating its association with nutritional status and positioning it as a practical, noninvasive measure of muscle function and strength decline related to malnutrition. The most important finding of our study was the development of an eight-parameter assessment model (a panel of gender, post-dialysis ECW/ICW ratio, post-dialysis lean body mass, post-dialysis MBF, serum albumin, nPCR, AC, and HGS) and the comparison of its diagnostic accuracy against SGA as the reference standard. This model demonstrated a clear advantage over the univariate PA model, having enhanced explanatory power, as confirmed by a higher pseudo-R^2^ value (0.46 vs. 0.23). The eight-parameter model also demonstrated very good discriminatory ability between malnourished and well-nourished individuals (AUC 0.88), significantly better than when considering PA alone in the univariate model. The panel of parameters also allowed the characterization of patients in terms of reduced muscle and fat mass, impaired muscle function, fluid shifts, metabolic dysfunction, and weakened cellular integrity. The practical application of the results may involve differentiating types of malnutrition, identifying patients with latent malnutrition, and supporting clinical decision-making and the implementation of timely nutritional intervention, which directly affects prognosis, quality of life, and hospitalization rate.

According to current recommendations, the assessment of muscle function (e.g., HGS) and body composition analysis are increasingly incorporated as elements of an integrated approach to clinical status evaluation [2,12,14]. Research by other authors has shown that biomarkers such as HGS, biochemical markers, anthropometric and bioimpedance measurements, with BIA increasingly being part of routine clinical and nutritional monitoring protocols in dialysis centers, may be particularly useful in ambiguous situations, or where comprehensive clinical assessment is not available [40]. Our attempt to verify the associations and diagnostic value of selected parameters with respect to the validated SGA not only aligns with current trends in nephrology and clinical dietetics, but may also contribute to improving clinical practice, particularly in the early detection of malnutrition and monitoring the effectiveness of nutritional interventions. The presented work emphasizes the importance of the SGA as a recognized tool in assessing nutritional status in HD patients. It is a widely used and recommended measure, and its clinical value has been confirmed in numerous studies [2,3,4,5,6,7,8]. However, incorporating simple, objective, and easily accessible measures such as HGS may increase diagnostic sensitivity, particularly in early-stage malnutrition or in overweight and obese patients, where classic malnutrition symptoms may be masked by adiposity.

Notably, PA, a bioimpedance parameter reflecting cellular quality and integrity [41,42], is an important indicator of poor prognosis and mortality, especially in elderly HD patients [43]. Low PA values (≤ 5.1°) indicate lost protein and energy reserves characteristic of PEW-related malnutrition [5]. Previous studies have demonstrated PA as the strongest predictor of malnutrition according to the SGA criterion [44], consistent with our findings. PA may enable the early detection of subclinical malnutrition [17,18], underscoring its utility in evaluating nutritional status, particularly in dialysis cohorts where BIA is feasible. However, our study revealed moderate sensitivity for PA, meaning limitations in detecting all cases and suggesting its use in combination with other measures rather than as a standalone screening tool, as a significant portion of malnourished patients could be missed. Similarly, HGS has been identified as a practical tool in current K/DOQI guidelines, consistent with GLIM criteria, which emphasize regular muscle function assessment as part of complete nutritional status evaluation [2]. Although established for sarcopenia, HGS is not widely used for malnutrition diagnosis, despite indicating its functional consequences even with normal body weight and often decreasing earlier than weight [45]. Malnutrition may impair muscle contraction and relaxation, which can affect HGS [46]. A large cohort study showed lower HGS significantly associated with higher malnutrition risk regardless of age, gender, diabetes, and CKD stage [45]. Başcı et al. found only HGS correlated with SGA, with higher HGS linked to increased lean mass ratio and AC but decreased fat tissue index [47]. Our study confirmed HGS as a simple indicator of complications risk, more sensitive than anthropometric measurements, though it may decrease independently of nutritional status, potentially weakening the relationship between HGS and malnutrition.

Similar to other authors, we confirmed that serum albumin should not be used as a standalone marker of malnutrition due to its susceptibility to confounding factors unrelated to nutrition such as inflammation and hydration status [2]. AC and CC are common methods helping in assessing nutritional status but are not standalone gold standards and should also be interpreted alongside other indicators, as demonstrated with AC in a large-scale study including diabetic patients [48] and smaller evaluation showing lower AC in malnourished individuals [46]. The frequent coexistence of diabetes in HD patients may justify the use of these parameters in the present study. BMI and fat-related parameters present additional limitations, as highlighted by the “obesity paradox” where higher BMI and fat tissue content in HD patients associate with lower mortality risk [46,49]. Similar to our findings, other studies have demonstrated that BMI-based criteria identify only a small proportion of malnourished patients, often underestimating its prevalence in HD patients. BMI is not considered a reliable indicator of body composition, as it does not distinguish between fat mass, muscle mass, and body water content [47,50,51]. Additionally, WHR has been linked to increased mortality risk [52,53] and VAI to the metabolically unhealthy nonobese (MUNO) phenotype in HD patients [54].

Fluid balance assessment is an important aspect in HD patient care, with our findings showing increased ECW index and ECW/ICW ratio as the degree of malnutrition increased according to the SGA criterion. The study by Ruperto and Barril emphasized combining methods for nutritional, obesity, and hydration assessments, introducing “sarcopenic obesity”—more prevalent in HD patients, where most were malnourished and overhydrated (as indicated by elevated ECW)—and other contributing factors including age, PA, and BMI [55]. Similarly, our study proved post-dialysis lean body mass to be a significant parameter, consistent with other reports emphasizing its and muscle mass’s importance as indicators of malnutrition risk. Direct body composition assessment allows the differentiation of lost muscle and fat reserves characteristic of different malnutrition phenotypes [56], which may proceed without obvious symptoms but associate with a high risk of complications. A higher ECW/ICW ratio indicates a tendency toward hydration imbalance and possible inflammatory state contributing to muscle mass loss, cellular atrophy, and deterioration of nutritional status [40,57]. The progression of malnutrition and sarcopenia is driven by increased catabolism and impaired protein synthesis, leading to lean body mass loss that contributes to fluid overload and disrupts post-dialysis fluid balance, fueling a vicious cycle of worsening malnutrition and muscle wasting. Chronic fluid overload places continuous hemodynamic stress on the heart, intensifying oxidative stress and myocardial inflammation. Additionally, muscle loss—an inflammatory process in itself—further accelerates the body’s deterioration. This condition, known as malnutrition–inflammation–fluid overload (MIFO) syndrome, is increasingly recognized as a hallmark of dialysis-related complications. Since fluid overload can worsen malnutrition, reducing fluid retention may be as crucial as nutritional support for improving outcomes [40].

Clinical indications from our study suggest that nutritional status assessment in HD patients should be comprehensive, as functional measures such as HGS and lean body mass, not only single indicators (e.g., PA, BMI, or serum albumin), were crucial in determining malnutrition likelihood. Our multivariable model emphasized combining muscle function and body composition in dialysis patients. Including HGS and lean body mass measurement can improve the early identification of individuals at risk of malnutrition and enable earlier nutritional and rehabilitation interventions. In the multivariable model, constructed based on clinical experience and previous research, even parameters that did not reach statistical significance may have clinical importance and affect the quality of the model prediction. Regular HGS measurements are simple, inexpensive, effective, and worth including in the routine assessment of HD patients, with added value in determining the risk of complications. Bioimpedance and body composition measurements (e.g., lean body mass) provide important information complementing classic biochemical parameters, potentially improving the identification of patients requiring intervention. Including parameters such as ECW/ICW ratio, serum albumin, and nPCR—although statistically non-significant in this model—when considered together with other indicators may support comprehensive clinical assessment, as omitting these biomarkers could overlook metabolic deterioration and nutritional deficiencies. The analysis indicates the clinical importance of individualized care, prioritizing patients with low muscle strength and lean body mass for nutritional and rehabilitation programs to improve outcomes. Male gender showing a trend toward greater risk of malnutrition may reflect biological differences, including distinct gender differences in skeletal muscle kinetics and fiber-type composition, as well as hormonal influences such as the anabolic effects of testosterone and anti-inflammatory effects of estrogen, affecting body composition and muscle function [58]. Muscle mass loss is often not reflected by changes in body weight or body mass indices, due to simultaneous increase in fat mass. Although the gender factor is non-modifiable, its presence in the model certainly supports the need to consider gender in nutritional prevention and monitoring plans, particularly in high-risk groups, to adapt care, vigilance, and intervention. Men typically have greater muscle mass and lose it quickly as CKD progresses. Catabolism also differs in men, and they may have different eating or physical activity patterns, report symptoms less frequently, and seek nutritional support less often.

This study is subject to several potential sources of bias. First, selection bias may have occurred due to the two-center design and voluntary participation, potentially excluding patients with more severe disease. Second, measurement error in BIA analysis may have occurred, since fluid retention in HD patients may affect accuracy by overestimating muscle mass. Third, HGS measurement bias may have occurred, as measurements were performed on the arm without arteriovenous fistula in order to minimize the risk of exerting pressure on the fistula-bearing arm. A native arteriovenous fistula was created at the most distal feasible site. In accordance with standard principles, the fistula was preferably created on the non-dominant arm, typically the left. However, in seven patients who had fistulas on their dominant arm, HGS measurements were performed on the non-dominant hand. This may have affected the results in a small proportion of participants (6.8%). Furthermore, assessment tools such as the SGA can also introduce observer bias. The risk of this bias was reduced because all examinations and measurements were performed by the same investigator, trained in this field. Additionally, confounding variables such as the duration of dialysis and comorbidities were not further examined and could have influenced both the indicators of nutritional status and body fat distribution. There were several limitations to this study. First, it involved a relatively small number of participants, which was due to the complexity of the methodology; therefore, the results cannot be generalized. Second, it was impossible in this study to establish a cause-and-effect relationship between malnutrition and the assessed parameters. The third limitation was related to the measurement of HGS. It was not possible to assess differences between patients using their dominant hand and those using their non-dominant hand, as HGS was measured on the hand without an arteriovenous fistula to avoid complications.

This study included patients from only two dialysis centers, both of which followed identical procedures. This facilitated the elimination of confounding factors associated with differences in standards and protocols. A strength of our study was that all measurements and laboratory tests were performed using the same method—on the same equipment. An added value of this study was the implementation of dietary education conducted by a clinical dietitian for our patients in conjunction with the nutritional status assessment. Future studies should validate and expand upon the proposed parameter panels to enhance their clinical applicability in dialysis patients. Future studies should also include multicenter cohorts with larger samples to identify optimal cutpoints for individual parameters, such as PA. Longitudinal studies are also needed to examine dynamic changes in these parameters as nutritional status changes. Furthermore, scientific data are lacking regarding how often bioimpedance testing should be performed in HD patients to effectively predict malnutrition risk. The finding regarding the relationship between gender and malnutrition also requires confirmation in HD patients. Given the observed borderline significance of gender as a predictor in the multivariable model, which indicates potential gender-specific differences in malnutrition risk among dialysis patients, further analysis is warranted to refine diagnostic approaches. Specifically, it is recommended to conduct gender-stratified evaluations to estimate optimal cutpoints for individual clinical parameters and to develop tailored multivariable panels separately for males and females. Such analyses should also incorporate larger sample sizes to mitigate power limitations and include validation through cross-validation or external cohorts to ensure generalizability. Expanding knowledge about complex associations among adiposity, functional status, and anthropometric measures with nutritional status has allowed for a deeper understanding of the multidimensional mechanisms leading to malnutrition. Analysis of these parameters revealed that malnutrition is not solely a consequence of reduced body mass but may also coexist with excessive adipose tissue (the so-called malnutrition with concurrent obesity), as well as reduced lean component and deterioration in functional status. An integrated approach that considers organism functionality and body composition allows for more precise development of effective preventive and therapeutic strategies, especially in populations of older HD patients and/or those with multiple comorbidities.

## 5. Conclusions

In conclusion, this study of associations of adiposity, functional status, and anthropometric measures with nutritional status indicated that the panel of eight parameters offered markedly superior discriminatory ability for identifying malnutrition and cachexia over PA alone, the best single predictor. The multifaceted nature of malnutrition requires comprehensive assessment, as even the best single predictor or metrics may not fully capture the condition’s complexity. Overall, the multivariable model’s superior performance advocates for a holistic diagnostic approach in clinical practice, integrating bioelectrical, functional, anthropometric, and biochemical markers.

## Figures and Tables

**Table 1:** Sociodemographic and clinical characteristics of patients participating in the study (*n* = 103) in relation to nutritional status.

Variable	Study Group Total *n* = 103	SGA-(B+C) *n* = 52 (50.5%)	SGA-A *n* = 51 (49.5%)	SGA-(B+C) vs. SGA-A
	M ± SD (95% CI) Me (IQR)	M ± SD (95% CI) Me (IQR)	M ± SD (95% CI) Me (IQR)	*p*-Value Cohen’s *d*
Age [years] *	61 ± 14 (59:64) 65 (51–71)	64 ± 14 (61:68) 66 (58–75)	58 ± 15 (54:63) 62 (50–70)	*p* = 0.044 *d* = 0.42
Gender, *n* (%)				
Male	64 (62.1%)	27 (51.9%)	37 (72.5%)	*p* = 0.031
Female	39 (37.9%)	25 (48.1%)	14 (27.5%)	
Education, *n* (%)				
Primary education	11 (10.7%)	8 (15.4%)	3 (5.9%)	
Vocational education	32 (31.1%)	15 (28.8%)	17 (33.3%)	
Secondary education	37 (35.9%)	19 (36.5%)	18 (35.3%)	
Higher education	23 (22.3%)	10 (19.2%)	13 (25.5%)	
Place of residence, *n* (%)				
Village	12 (11.7%)	8 (15.4%)	4 (7.8%)	
Small town	7 (6.8%)	1 (1.9%)	6 (11.8%)	
Medium-sized town	13 (12.6%)	6 (11.5%)	7 (13.7%)	
Large city	71 (68.9%)	37 (71.2%)	34 (66.7%)	
Marital status, *n* (%)				
Single	17 (16.5%)	8 (15.4%)	9 (17.6%)	
Married	59 (57.3%)	28 (53.8%)	31 (60.8%)	
Divorced	9 (8.7%)	3 (5.8%)	6 (11.8%)	
Widow/Widower	18 (17.5%)	13 (25.0%)	5 (9.8%)	
Social status, *n* (%)				
Unemployed	6 (5.8%)	2 (3.8%)	4 (7.8%)	
Employee	16 (15.5%)	6 (11.5%)	10 (19.6%)	
Retiree/Pensioner	81 (78.6%)	44 (84.6%)	37 (72.5%)	
Vascular access, *n* (%)				
Natural arteriovenous fistula	70 (68.0%)	38 (73.1%)	32 (62.7%)	
Permanent catheter	33 (32.0%)	14 (26.9%)	19 (37.3%)	
Presence of residual diuresis, *n* (%)	82 (79.6%)	41 (78.8%)	41 (80.4%)	
Hospitalization within the last year, *n* (%)	67 (65.0%)	33 (63.5%)	34 (66.7%)	
Cause of hospitalization, *n* (%) **				
COVID-19	7 (10.4%)	3 (9.1%)	4 (11.8%)	
Vascular access issues	38 (56.7%)	17 (51.5%)	21 (61.8%)	
Cardiovascular diseases	12 (17.9%)	7 (21.2%)	5 (14.7%)	
Others	26 (38.8%)	15 (45.5%)	11 (32.4%)	
Cause of CKD/comorbidities, *n* (%) **				
Diabetic kidney disease	25 (24.3%)	10 (19.2%)	15 (29.4%)	
Hypertensive nephropathy	52 (50.5%)	24 (46.2%)	28 (54.9%)	
Chronic glomerulonephritis	25 (24.3%)	15 (28.8%)	10 (19.6%)	
Others	76 (73.8%)	35 (67.3%)	41 (80.4%)	
Dialysis vintage [months] *	65 ± 69 (51:79) 42 (15–82)	71 ± 76 (50:92) 45 (13–103)	59 ± 62 (41:76) 41 (19–78)	*p* = 0.826 *d* = 0.18
Residual diuresis [mL] *	616.5 ± 616.5 (496.0:737.0) 500.0 (100.0–1000.0)	551.9 ± 593.3 (386.8:717.1) 500.0 (100.0–875.0)	682.4 ± 638.3 (502.8:861.9) 500.0 (100.0–1000.0)	*p* = 0.335 *d* = −0.21
UFV [L]	2.2 ± 1.0 (2.0:2.4) 2.2 (1.3–3.1)	2.1 ± 1.0 (1.9:2.4) 2.1 (1.4–2.9)	2.2 ± 1.1 (1.9:2.5) 2.4 (1.1–3.2)	*p* = 0.954 *d* = −0.05
Ideal body weight [kg]	66.5 ± 10.6 (64.4:68.6) 67.5 (58.2–73.9)	64.8 ± 11.7 (61.5:68.0) 64.0 (56.9–72.7)	68.2 ± 9.2 (65.6:70.8) 69.0 (62.2–74.3)	*p* = 0.151 *d* = −0.33
Height [cm]	166.6 ± 10.4 (164.6:168.6) 167.5 (159.4–174.0)	165.0 ± 11.3 (161.8:168.1) 164.0 (157.4–173.4)	168.2 ± 9.2 (165.6:170.8) 169.0 (162.2–174.3)	*p* = 0.166 *d* = −0.32
BMI [kg/m^2^] *	27.1 ± 6.6 (25.8:28.4) 26.0 (22.7–30.4)	25.6 ± 6.6 (23.8:27.5) 24.7 (21.0–29.1)	28.6 ± 6.4 (26.8:30.4) 27.7 (24.1–32.7)	*p* = 0.012 *d* = −0.46
% Overweight *	18.8 ± 23.8 (14.2:23.5) 8.7 (0.0–28.7)	15.6 ± 22.0 (9.5:21.8) 5.4 (0.0–22.6)	22.1 ± 25.3 (15.0:29.2) 13.8 (2.3–33.6)	*p* = 0.083 *d* = −0.27
BAI *	27.7 ± 6.4 (26.5:29.0) 26.9 (23.2–31.8)	27.4 ± 7.0 (25.5:29.4) 26.5 (23.1–31.6)	28.0 ± 5.7 (26.4:29.6) 27.2 (23.2–31.9)	*p* = 0.617 *d* = −0.09
VAI *	2.97 ± 2.58 (2.45:3.49) 2.11 (1.25–3.91)	2.70 ± 2.20 (2.07:3.32) 2.03 (1.05–3.71)	3.25 ± 2.93 (2.40:4.10) 2.17 (1.26–4.25)	*p* = 0.249 *d* = −0.21
WHR	0.99 ± 0.11 (0.96:1.01) 0.97 (0.89–1.06)	0.98 ± 0.11 (0.95:1.01) 0.95 (0.89–1.07)	0.99 ± 0.11 (0.96:1.02) 1.00 (0.92–1.06)	*p* = 0.753 *d* = −0.09
Waist circumference [cm] *	96.7 ± 17.9 (93.2:100.2) 93.5 (82.5–110.0)	94.1 ± 18.3 (89.0:99.2) 91.0 (80.0–110.0)	99.4 ± 17.2 (94.5:104.2) 99.0 (86.0–111.0)	*p* = 0.113 *d* = −0.30
HC [cm] *	97.7 ± 12.0 (95.4:100.1) 97.0 (91.5–103.0)	95.6 ± 13.1 (91.9:99.2) 94.0 (89.0–100.5)	99.9 ± 10.3 (97.0:102.8) 97.0 (94.0–104.0)	*p* = 0.013 *d* = −0.37
AC [cm] *	28.9 ± 4.4 (28.1:29.8) 29.0 (26.0–31.0)	27.4 ± 4.2 (26.2:28.6) 28.0 (23.8–30.0)	30.5 ± 4.1 (29.3:31.6) 30.0 (28.0–33.0)	*p* = 0.001 *d* = −0.74
CC [cm] *	35.8 ± 4.5 (35.0:36.7) 36.0 (33.5–38.5)	34.8 ± 4.6 (33.5:36.1) 35.3 (32.0–37.0)	36.9 ± 4.2 (35.7:38.1) 36.5 (35.0–40.0)	*p* = 0.003 *d* = −0.48
Wrist circumference [cm] *	18.5 ± 3.2 (17.8:19.1) 18.0 (17.0–19.5)	18.0 ± 1.9 (17.4:18.5) 18.0 (16.3–19.5)	19.0 ± 4.1 (17.8:20.1) 18.5 (17.5–19.5)	*p* = 0.178 *d* = −0.32
HGS [kg] *	28.6 ± 11.9 (26.3:31.0) 27.3 (19.4–36.1)	25.1 ± 13.1 (21.4:28.8) 20.9 (15.4–32.8)	32.3 ± 9.4 (29.6:34.9) 33.2 (24.8–39.8)	*p* < 0.001 *d* = −0.63
URR (%) *	74.6 ± 6.1 (73.4:75.8) 75.2 (70.6–78.1)	75.6 ± 6.9 (73.7:77.5) 76.8 (71.6–80.4)	73.6 ± 5.0 (72.2:75.1) 74.3 (70.0–77.1)	*p* = 0.036 *d* = 0.32
Pre-dialysis urea concentration [mmol/L] *	19.3 ± 5.6 (18.2:20.4) 18.7 (16.0–22.0)	18.3 ± 4.8 (17.0:19.7) 18.5 (15.7–20.9)	20.3 ± 6.1 (18.6:22.0) 20.2 (16.4–22.6)	*p* = 0.067 *d* = −0.37
Post-dialysis urea concentration [mmol/L] *	5.0 ± 2.2 (4.6:5.4) 4.7 (3.6–6.1)	4.7 ± 2.5 (4.0:5.3) 4.1 (3.4–5.4)	5.4 ± 1.9 (4.8:5.9) 5.1 (4.3–6.6)	*p* = 0.007 *d* = −0.33
Post-dialysis body weight [kg] *	75.3 ± 20.2 (71.3:79.2) 74.9 (64.3–85.4)	70.0 ± 20.4 (64.3:75.6) 69.6 (58.3–78.6)	80.7 ± 18.7 (75.5:86.0) 78.4 (67.4–86.8)	*p* = 0.002 *d* = −0.55
Post-dialysis protein [kg]	9.7 ± 2.4 (9.3:10.2) 9.5 (8.1–11.1)	9.0 ± 2.3 (8.3:9.6) 8.6 (7.1–10.7)	10.5 ± 2.2 (9.9:11.2) 10.5 (8.7–12.2)	*p* < 0.001 *d* = −0.71
Post-dialysis SMM [kg]	27.3 ± 7.1 (25.9:28.7) 26.6 (22.5–31.5)	24.8 ± 6.8 (22.9:26.7) 23.6 (19.2–29.8)	29.8 ± 6.6 (27.9:31.7) 29.4 (24.3–34.9)	*p* < 0.001 *d* = −0.74
Post-dialysis lean body mass [kg]	50.6 ± 11.6 (48.3:52.8) 49.0 (41.4–57.6)	47.2 ± 11.3 (44.0:50.3) 45.5 (37.7–55.6)	54.0 ± 11.0 (50.9:57.1) 53.3 (45.2–63.0)	*p* = 0.005 *d* = −0.61
Post-dialysis MBF [kg] *	24.9 ± 14.7 (22.1:27.8) 22.4 (13.4–33.9)	23.2 ± 14.7 (19.1:27.3) 22.1 (12.1–30.9)	26.7 ± 14.7 (22.6:30.9) 23.3 (15.2–38.1)	*p* = 0.164 *d* = −0.24
Post-dialysis fat tissue content (%)	31.5 ± 12.7 (29.0:33.9) 31.8 (21.0–42.0)	31.1 ± 13.1 (27.4:34.8) 30.7 (21.9–41.8)	31.8 ± 12.4 (28.3:35.3) 31.9 (20.5–42.8)	*p* = 0.820 *d* = −0.05
Post-dialysis extracellular water index	0.391 ± 0.016 (0.388:0.394) 0.391 (0.379–0.403)	0.398 ± 0.014 (0.394:0.402) 0.400 (0.388–0.407)	0.384 ± 0.014 (0.380:0.388) 0.386 (0.373–0.395)	*p* < 0.001 *d* = 1.05
Post-dialysis extracellular water [L]	14.4 ± 3.3 (13.8:15.1) 14.4 (11.8–16.6)	13.7 ± 3.4 (12.8:14.6) 13.7 (11.1–16.6)	15.1 ± 3.0 (14.3:16.0) 14.9 (12.8–17.2)	*p* = 0.039 *d* = −0.45
Post-dialysis intracellular water [L]	22.5 ± 5.5 (21.5:23.6) 22.0 (18.8–25.7)	20.7 ± 5.3 (19.2:22.2) 19.8 (16.3–24.7)	24.4 ± 5.0 (23.0:25.8) 24.1 (20.2–28.3)	*p* < 0.001 *d* = −0.71
Post-dialysis ECW/ICW ratio	0.643 ± 0.043 (0.635:0.652) 0.641 (0.611–0.672)	0.663 ± 0.039 (0.652:0.674) 0.665 (0.635–0.687)	0.623 ± 0.037 (0.613:0.634) 0.628 (0.594–0.652)	*p* < 0.001 *d* = 1.05
Post-dialysis PA [°]	5.65 ± 1.38 (5.38:5.91) 5.60 (4.70–6.60)	4.97 ± 1.17 (4.65:5.30) 5.00 (4.20–5.75)	6.33 ± 1.23 (5.98:6.68) 6.10 (5.50–7.00)	*p* < 0.001 *d* = −1.13
Creatinine [mg/dL] *	8.89 ± 2.56 (8.38:9.39) 9.00 (7.20–10.50)	7.94 ± 2.15 (7.33:8.55) 8.03 (6.26–9.40)	9.81 ± 2.62 (9.07:10.55) 9.85 (8.46–11.70)	*p* < 0.001 *d* = −0.78
Albumin [g/L] *	38.5 ± 4.0 (37.7:39.2) 39.0 (37.0–41.0)	37.3 ± 4.6 (36.0:38.6) 37.6 (35.8–40.0)	39.6 ± 2.8 (38.8:40.4) 40.0 (38.0–41.6)	*p* = 0.001 *d* = −0.60
Total protein [g/L]	65.6 ± 4.6 (64.7:66.6) 65.8 (63.1–68.4)	65.5 ± 4.6 (64.2:66.8) 65.2 (63.0–68.6)	65.8 ± 4.6 (64.5:67.2) 65.8 (63.2–67.9)	*p* = 0.692 *d* = −0.08
Transferrin [g/L] *	1.74 ± 0.33 (1.67:1.80) 1.74 (1.49–1.92)	1.69 ± 0.33 (1.59:1.78) 1.68 (1.45–1.91)	1.79 ± 0.32 (1.69:1.88) 1.75 (1.55–1.93)	*p* = 0.161 *d* = −0.31
Total cholesterol [mmol/L] *	4.32 ± 1.24 (4.07:4.57) 3.99 (3.46–5.15)	4.27 ± 1.21 (3.93:4.61) 3.86 (3.47–4.91)	4.38 ± 1.27 (4.01:4.75) 4.07 (3.45–5.18)	*p* = 0.459 *d* = −0.09
HDL [mmol/L] *	1.11 ± 0.47 (1.02:1.20) 1.01 (0.82–1.31)	1.12 ± 0.47 (0.99:1.26) 1.03 (0.82–1.31)	1.10 ± 0.46 (0.97:1.23) 0.98 (0.82–1.29)	*p* = 0.583 *d* = 0.05
LDL [mmol/L] *	2.60 ± 1.04 (2.39:2.81) 2.40 (1.86–3.25)	2.60 ± 1.07 (2.30:2.90) 2.40 (1.85–3.18)	2.60 ± 1.02 (2.30:2.90) 2.39 (1.86–3.26)	*p* = 0.941 *d* = 0.00
TG [mmol/L] *	1.65 ± 1.06 (1.43:1.86) 1.41 (0.85–1.98)	1.48 ± 0.89 (1.23:1.73) 1.37 (0.82–1.79)	1.82 ± 1.20 (1.47:2.17) 1.50 (0.94–2.50)	*p* = 0.193 *d* = −0.32
hs-CRP [mg/L] *	8.8 ± 14.3 (6.0:11.7) 4.3 (1.4–9.2)	10.7 ± 18.1 (5.6:15.8) 4.4 (2.2–9.3)	6.9 ± 8.3 (4.5:9.3) 4.2 (1.2–9.1)	*p* = 0.412 *d* = 0.27
nPCR [g/kg/day]	1.02 ± 0.23 (0.98:1.07) 1.02 (0.87–1.16)	1.00 ± 0.19 (0.95:1.05) 1.02 (0.85–1.14)	1.05 ± 0.25 (0.98:1.12) 1.03 (0.89–1.20)	*p* = 0.406 *d* = −0.22

Note: Results are the M ± SD (and 95% CI for the mean) and Me (25–75th percentiles) for continuous variables or numbers (and percentages) for categorical variables. * Non-normally distributed data (Shapiro–Wilk test, *p* < 0.05). ** Some patients experienced more than one event. Abbreviations: *n*—number of participants; M—arithmetic mean; SD—standard deviation; CI—confidence interval; Me—median; IQR—interquartile range; *p*—probability testing; *d*—Cohen’s *d* effect size, *d* < 0 indicates lower values in SGA-(B + C); COVID-19—coronavirus disease 2019; CKD—chronic kidney disease; UFV—ultrafiltration volume; BMI—body mass index; BAI—body adiposity index; VAI—visceral adiposity index; WHR—waist-to-hip ratio; HC—hip circumference; AC—arm circumference; CC—calf circumference; HGS—handgrip strength; URR—urea reduction ratio; SMM—skeletal muscle mass; MBF—mass of body fat (body fat mass); ECW/ICW—extracellular to intracellular water (extracellular water/intracellular water); PA—phase angle; HDL—high-density lipoprotein; LDL—low-density lipoprotein; TG—triglycerides; hs-CRP—high-sensitivity *C*-reactive protein; nPCR—normalized protein catabolic rate.

**Table 2:** Associations of analyzed variables according to selected clinical cutoff points in relation to nutritional status (SGA-B + C vs. SGA-A) in studied patients (*n* = 103).

Variable		SGA-(B+C), *n* (%)	SGA-A, *n* (%)	χ^2^; *df* = 1; *p*-Value; Kendall’s *τ*_b_; Cohen’s κ
		*n* = 52 (50.5%)	*n* = 51 (49.5%)	
HGS ^†^	≤Cutoff point, *n* = 33 (32.0%)	22 (66.7%)	11 (33.3%)	χ^2^ = 4.18; *p* = 0.041; *τ*_b_ = 0.22; κ = 0.207
	>Cutoff point, *n* = 70 (68.0%)	30 (42.9%)	40 (57.1%)	
BMI	<23 kg/m^2^, *n* = 28 (27.2%)	19 (67.9%)	9 (32.1%)	χ^2^ = 3.74; *p* = 0.053; *τ*_b_ = 0.21; κ = 0.188
	≥23 kg/m^2^, *n* = 75 (72.8%)	33 (44.0%)	42 (56.0%)	
WHR ^‡^	Visceral fat tissue within normal range, *n* = 26 (25.2%)	10 (38.5%)	16 (61.5%)	χ^2^ = 1.42; *p* = 0.233; *τ*_b_ = −0.14; κ = −0.121
	Visceral obesity, *n* = 77 (74.8%)	42 (54.5%)	35 (45.5%)	
AC	≤22 cm, *n* = 8 (7.8%)	8 (100.0%)	0 (0.0%)	χ^2^ = 6.49; *p* = 0.011; *τ*_b_ = 0.29; κ = 0.153
	>22 cm, *n* = 95 (92.2%)	44 (46.3%)	51 (53.7%)	
CC	≤31 cm, *n* = 12 (11.7%)	10 (83.3%)	2 (16.7%)	χ^2^ = 4.47; *p* = 0.035; *τ*_b_ = 0.24; κ = 0.152
	>31 cm, *n* = 91 (88.4%)	42 (46.2%)	49 (53.8%)	
Post-dialysis fat tissue content	<10%, *n* = 6 (5.8%)	4 (66.7%)	2 (33.3%)	χ^2^ = 0.16; *p* = 0.692; *τ*_b_ = 0.08; κ = 0.037
	≥10%, *n* = 97 (94.2%)	48 (49.5%)	49 (50.5%)	
Post-dialysis PA	≤5°, *n* = 33 (32.0%)	27 (81.8%)	6 (18.2%)	χ^2^ = 17.27; *p* < 0.001; *τ*_b_ = 0.43; κ = 0.400
	>5°, *n* = 70 (68.0%)	25 (35.7%)	45 (64.3%)	

^†^  HGS cutoff points for low muscle strength dependent on gender and BMI: women—BMI ≤ 23: ≤17 kg, BMI 23.1–26: ≤17.3 kg, BMI 26.1–29: ≤18 kg, BMI > 29: ≤21 kg; men—BMI ≤ 24: ≤29 kg, BMI 24.1–28: ≤30 kg, BMI > 28: ≤32 kg. ^‡^ WHR < 0.8 (women), WHR < 1 (men)—visceral fat tissue within normal range; WHR ≥ 0.8 (women), WHR ≥ 1 (men)—visceral obesity. Abbreviations: SGA—Subjective Global Assessment; χ^2^—chi-square test with Yates’ correction; *τ*_b_—Kendall’s tau-b coefficient; κ—Cohen’s kappa coefficient.

**Table 3:** The basic descriptive statistics for adiposity and selected bioelectrical impedance parameters, along with Spearman’s rank correlation with the SGA.

Variable	*n* = 103	SGA and Variable	
	M ± SD (95% CI)	*ρ*	*p*
BAI	27.7 ± 6.4 (26.5:29.0)	−0.08	0.418
VAI	2.97 ± 2.58 (2.45:3.49)	−0.14	0.158
AC [cm]	28.9 ± 4.4 (28.1:29.8)	−0.38	<0.001
CC [cm]	35.8 ± 4.5 (35.0:36.7)	−0.36	<0.001
HGS [kg]	28.6 ± 11.9 (26.3:31.0)	−0.34	<0.001
Post-dialysis body weight [kg]	75.3 ± 20.2 (71.3:79.2)	−0.38	<0.001
Post-dialysis SMM [kg]	27.3 ± 7.1 (25.9:28.7)	−0.39	<0.001
Post-dialysis lean body mass [kg]	50.6 ± 11.6 (48.3:52.8)	−0.32	0.001
Post-dialysis MBF [kg]	24.9 ± 14.7 (22.1:27.8)	−0.21	0.037
Post-dialysis fat tissue content (%)	31.5 ± 12.7 (29.0:33.9)	−0.08	0.448
Post-dialysis extracellular water index	0.391 ± 0.016 (0.388:0.394)	0.39	<0.001
Post-dialysis extracellular water [L]	14.4 ± 3.3 (13.8:15.1)	−0.26	0.007
Post-dialysis intracellular water [L]	22.5 ± 5.5 (21.5:23.6)	−0.37	<0.001
Post-dialysis ECW/ICW ratio	0.643 ± 0.043 (0.635:0.652)	0.39	<0.001
Post-dialysis PA [°]	5.65 ± 1.38 (5.38:5.91)	−0.46	<0.001
Albumin [g/L]	38.5 ± 4.0 (37.7:39.2)	−0.32	0.004

*p* < 0.05. Abbreviation: *ρ*—Spearman’s rank correlation coefficient.

**Table 4:** Optimal cutpoints for parameters in diagnosing malnutrition (including cachexia) compared to normal nutritional status.

Parameter	Optimal Threshold	Youden’s Index	Accuracy	Sensitivity	Specificity	AUC	95% CI	Prevalence
BMI	≤23.58 kg/m^2^	0.27	0.63	0.44	0.82	0.64	0.49:0.78	0.50
BAI *	≤25.34	0.08	0.54	0.39	0.69	0.53	0.38:0.67	0.50
VAI	≤2.65	0.19	0.59	0.56	0.63	0.57	0.41:0.72	0.51
Post-dialysis ECW/ICW ratio *	≥0.66	0.40	0.70	0.54	0.86	0.77	0.63:0.88	0.50
Post-dialysis PA *	≤5.1°	0.46	0.73	0.58	0.88	0.79	0.66:0.89	0.50
Post-dialysis SMM	≤21.0 kg	0.25	0.62	0.40	0.84	0.60	0.44:0.75	0.50
Post-dialysis lean body mass	≤39.8 kg	0.31	0.65	0.37	0.94	0.66	0.51:0.80	0.50
Post-dialysis MBF	≤35.3 kg	0.20	0.60	0.88	0.31	0.58	0.42:0.72	0.50
Albumin	≤37.3 g/L	0.30	0.65	0.50	0.80	0.69	0.53:0.82	0.50
nPCR	≤1.19 g/kg/day	0.16	0.58	0.88	0.27	0.54	0.38:0.69	0.50
AC	≤25.5 cm	0.29	0.64	0.35	0.94	0.68	0.53:0.82	0.50
CC	≤37.5 cm	0.30	0.65	0.87	0.43	0.68	0.52:0.81	0.50
HGS	≤23.3 kg	0.43	0.71	0.59	0.84	0.71	0.58:0.85	0.50
WHR	≤0.95	0.19	0.59	0.56	0.63	0.54	0.38:0.69	0.50
Gender **	—	—	0.60	0.48	0.73	0.60	0.51:0.70	0.50

Notes: Optimal thresholds were determined with the maximize metric method, optimizing Youden’s index (Youden’s index = sensitivity + specificity − 1). The direction “≥” indicates that values equal to or greater than the threshold are classified as positive (malnutrition or cachexia); “≤” indicates that values equal to or less than the threshold are classified as positive. Prevalence—proportion of the positive class in the dataset. * Multiple optimal cutpoints identified; tie-breaking applied. ** Categorical variable with female as positive class with only performance metrics being estimated. Abbreviation: AUC—area under the receiver operating characteristic curve.

**Table 5:** Comparative effects of logistic regression models: multivariable panel versus best univariate predictor (PA) in predicting malnutrition.

Predictor	Multivariable Model			Univariate Model		
	OR	95% CI	*p*	OR	95% CI	*p*
(Intercept)	0.01	0.00–0.12	<0.001	0.49	0.29–0.80	0.006
PA [≤5.1° vs. >5.1°]	—	—	—	10.23	3.93–30.61	<0.001
Gender [Male vs. Female]	5.48	0.90–33.48	0.061	—	—	—
Post-dialysis ECW/ICW ratio [≥0.66 vs. <0.66]	2.41	0.77–7.52	0.142	—	—	—
Post-dialysis lean body mass [≤39.8 kg vs. >39.8 kg]	6.65	1.20–36.89	0.029	—	—	—
Post-dialysis MBF [≤35.3 kg vs. >35.3 kg]	2.93	0.80–10.77	0.099	—	—	—
Albumin [≤37.3 g/L vs. >37.3 g/L]	2.49	0.85–7.29	0.108	—	—	—
nPCR [≤1.19 g/kg/day vs. >1.19 g/kg/day]	2.47	0.68–8.98	0.173	—	—	—
AC [≤25.5 cm vs. >25.5 cm]	5.18	0.87–30.89	0.064	—	—	—
HGS [≤23.3 kg vs. >23.3 kg]	7.54	1.50–37.90	0.011	—	—	—
Observations	103			103		
Pseudo-R^2^	0.460			0.232		

Abbreviation: OR—odds ratio.

## Data Availability

The data presented in this study are available on request from the corresponding author due to ethical reasons and protection of personal data.

## Supplementary Materials

The following supporting information can be downloaded at: https://www.mdpi.com/article/10.3390/nu17193034/s1, Supplemental Methods; Figure S1: The receiver operating characteristic (ROC) curve for the modeled probability of BMI < 23 kg/m^2^ based on the BAI and VAI; Table S1: Comparison of anthropometric measurements and HGS with fat distribution indices and BIA-derived parameters; Table S2: The logistic regression for the BMI variable, where the modeled variable was BMI < 23 kg/m^2^ based on the BAI and VAI variables; Table S3: The observed frequencies for the associations of analyzed variables; Table S4: Sociodemographic and clinical characteristics of patients participating in the study (*n* = 103) in relation to gender.

## Abbreviations

The following abbreviations are used in this manuscript:

AC	Arm circumference
AIC	Akaike information criterion
arctan	Arcus tangent (trigonometric function)
AUC	Area under the receiver operating characteristic curve
BAI	Body adiposity index
BIA	Bioelectrical impedance analysis
BMI	Body mass index
*C*_0_	Urea concentration before the hemodialysis session
*C_t_*	Urea concentration after the hemodialysis session
CC	Calf circumference
CI	Confidence interval
CKD	Chronic kidney disease
COVID-19	Coronavirus disease 2019
*d*	Cohen’s *d* effect size
DEI	Daily energy intake
DPI	Daily protein intake
EBPG	European Best Practice Guidelines on Nutrition
ECW	Extracellular water
ECW/ICW	Extracellular to intracellular water (extracellular water/intracellular water)
ECW/TBW	Extracellular water/total body water
EWGSOP	European Working Group on Sarcopenia in Older People
GLIM	Global Leadership Initiative on Malnutrition
GNRI	Geriatric Nutritional Risk Index
HC	Hip circumference
HD	Hemodialysis
HDL	High-density lipoprotein
HGS	Handgrip strength
hs-CRP	High-sensitivity *C*-reactive protein
ICW	Intracellular water
IQR	Interquartile range
ISRNM	International Society of Renal Nutrition and Metabolism
K/DOQI	Kidney Disease Outcomes Quality Initiative
*Kt*/*V*	Dialysis adequacy index
LDL	Low-density lipoprotein
M	Arithmetic mean
MIFO	Malnutrition–inflammation–fluid overload
MIS	Malnutrition-Inflammation Score
MNA	Mini Nutritional Assessment
MAMC	Mid-arm muscle circumference
MBF	Mass of body fat (body fat mass)
Me	Median
MUNO	Metabolically unhealthy nonobese
*n*	Number of participants
nPCR	Normalized protein catabolic rate
NYHA	New York Heart Association
OR	Odds ratio
*p*	Probability testing
PA	Phase angle
PEM	Protein–energy malnutrition
PEW	Protein–energy wasting
*R*	Resistance (tissue electrical resistance)
ROC	Receiver operating characteristic
SD	Standard deviation
SGA	Subjective Global Assessment
SMM	Skeletal muscle mass
STROBE	Strengthening the Reporting of Observational Studies in Epidemiology
TG	Triglycerides
UFV	Ultrafiltration volume
URR	Urea reduction ratio
VAI	Visceral adiposity index
VIF	Variance inflation factor
WHR	Waist-to-hip ratio
*X_c_*	Reactance
κ	Cohen’s kappa coefficient
*ρ*	Spearman’s rank correlation coefficient
*τ*_b_	Kendall’s tau-b coefficient
χ^2^	Chi-square test with Yates’ correction
