# Supplementary material for: Evaluating the Associations of Adiposity, Functional Status, and Anthropometric Measures with Nutritional Status in Chronic Hemodialysis Patients: A Cross-Sectional Study
Source: Nutrients. 2025 Sep 23;17(19):3034. doi: 10.3390/nu17193034 (PMC12526302; doi:10.3390/nu17193034)
Supplement: Supplementary file 1 [file nutrients-17-03034-s001.zip › nutrients-3875949-supplementary.pdf]

nutrients

MDPI

Article

Evaluating the Associations of Adiposity, Functional Status, and Anthropometric Measures with Nutritional Status in Chronic Hemodialysis Patients: A Cross-Sectional Study

Martyna Andreew-Gamza and Beata Hornik

Supplemental Methods

Additional Exclusion Criteria

Additional exclusion criteria related to body composition analysis using bioelectrical impedance included: pregnancy, epilepsy, the presence of a pacemaker or implantable cardioverter–defibrillator (ICD), and metal implants in the body. Additional exclusion criteria for handgrip strength (HGS) measurement using a dynamometer: body temperature > 38°C; functional limitations restricting additional physical activity; cardiovascular instability (including pharmacologically uncontrolled hypertension, pharmacologically difficult-to-control arrhythmia, angina at rest or during minimal exertion); heart failure > NYHA class II; severe valvular heart disease; serum potassium concentration > 6 mmol/L; advanced renal osteodystrophy; advanced retinopathy; patients with poorly functioning vascular access (arteriovenous fistula/catheter); and hemodynamic instability during dialysis (e.g., intradialytic hypotension, interdialytic weight gain > 6 L).

Sample Size

The study sample size n_{min} was calculated using Cochran's sample size formula for a known population size [20]. First, n_{min0} was calculated using the following formula:

$$n_{min_0} = \frac{t^2 P(1-P)}{e^2},$$

where t is the abscissa of the normal curve cutting off an area of $\alpha/2$ in each tail, P is the estimated population portion, and e is the margin of error. For the significance level α equal to 0.05, the critical value t is adopted as 1.96. According to the formula, the sample size is 384 at a 95% confidence level with an estimated population proportion of 0.5 and an acceptable sampling error of 0.05.

In this study, the population size was N = 113 (Figure 1), which was relatively not large, and the n_{min0}/N ratio in the formula below was not negligible. Therefore, the final sample size n_{min} was obtained using Cochran's correction formula for finite population size:

$$n_{min} = \frac{n_{min_0}}{1 + \frac{n_{min_0}}{N}}.$$

Supplemental Results

An additional analysis was conducted comparing patients based on their HGS values, dividing them into: those with normal HGS and those with reduced HGS (below the accepted norm). It should be clearly emphasized that this division was not based on

 https://doi.org/10.3390/xxxxx

nutritional status assessment according to the Subjective Global Assessment (SGA), but solely on muscle function. As presented in Table S1, patients with reduced HGS [kg], compared to those with HGS within the normal range, had significantly higher values for the following parameters: body adiposity index (BAI) value (29.6 ± 5.8 vs. 27.0 ± 6.4; $p = 0.049$); post-dialysis fat tissue content (36.5 ± 10.6% vs. 29.1 ± 13.0%; $p = 0.006$); post-dialysis extracellular water (ECW) index (0.40 ± 0.01 vs. 0.39 ± 0.02; $p = 0.003$); and extracellular to intracellular water (ECW/ICW) ratio (0.66 ± 0.04 vs. 0.64 ± 0.04; $p = 0.004$). In contrast, these patients had significantly lower values of post-dialysis skeletal muscle mass (SMM) [kg] (23.53 ± 5.48 kg vs. 29.07 ± 7.14 kg; $p < 0.001$); post-dialysis lean body mass [kg] (44.10 ± 9.29 kg vs. 53.61 ± 11.41 kg; $p < 0.001$); post-dialysis intracellular water (ICW) [L] (19.57 ± 4.20 L vs. 23.91 ± 5.44 L; $p = 0.001$); and post-dialysis phase angle (PA) [°] (4.90 ± 1.15° vs. 6.00 ± 1.34°; $p < 0.001$).

Cohen's *d* indicated varying effect sizes between groups with reduced and normal HGS. Large effects were observed for SMM ($d = -0.83$), lean body mass ($d = -0.88$), and PA ($d = -0.86$), indicating pronounced differences in these parameters. Moderate effects were found for fat tissue content ($d = 0.60$) and BAI ($d = 0.42$), while some parameters, such as visceral adiposity index (VAI), showed a negligible effect (Table S1).

In the present study, a decrease in HGS was associated with higher values of the following parameters: the BAI, fat tissue content, ECW, and ECW/ICW ratio, as well as lower values of parameters determined through body composition analysis, such as SMM, lean body mass, ICW, and PA. Effect size analysis provided deeper insight into the magnitude of differences between patients with reduced and normal HGS. Large effects ($d \geq 0.8$) were observed for muscle mass-related parameters and PA, emphasizing their critical role in assessing nutritional status and muscle function. Moderate effects for fat-related measures suggest that patients with reduced HGS may have increased adiposity, potentially reflecting sarcopenic obesity. Other studies have observed that hemodialysis (HD) patients tend to have low muscle mass and high fat mass content [47].

Similarly, in studies by other authors involving patients with chronic kidney disease (CKD) who were being prepared for kidney transplantation, HGS was assessed in combination with bioelectrical impedance analysis (BIA) parameters, as in the study by Michou et al. [27]. In our observations, an increase in HGS was associated with an increase in PA and the lean body mass, as determined through BIA. Other studies also measured the ECW and ICW indicators. It was observed that, as HGS decreased, the extracellular water/total body water (ECW/TBW) ratio and fat index increased, while the muscle mass index decreased [57].

Patients classified based on the body mass index (BMI) [kg/m²] criterion below the cutoff point (BMI < 23 kg/m²), compared to patients with BMI ≥ 23 kg/m², had significantly lower values of the following parameters: BAI (22.66 ± 4.22 vs. 29.71 ± 5.88; $p < 0.001$); VAI (2.05 ± 1.41 vs. 4.55 ± 3.92; $p = 0.002$); post-dialysis fat mass (9.95 ± 4.72 kg vs. 30.54 ± 13.22 kg; $p < 0.001$); and post-dialysis fat tissue content (17.58 ± 8.51% vs. 36.63 ± 9.84%; $p < 0.001$).

The comparison between patients with BMI < 23 kg/m² and those with BMI ≥ 23 kg/m² revealed large to very large effect sizes (Cohen's $d \geq 0.8$) for most adiposity-related parameters. The strongest differences were observed for fat tissue content ($d = -2.00$), body fat mass ($d = -1.78$), and BAI ($d = -1.29$), indicating notably lower fat reserves in patients with lower BMI. Body weight also showed a large effect ($d = -1.40$). In contrast, muscle mass and body water compartments demonstrated small to negligible differences (e.g., SMM: $d = -0.26$; ECW/ICW ratio: $d = 0.22$; PA: $d = -0.28$), suggesting that BMI primarily differentiated patients by fat mass rather than lean body mass or cellular health (Table S1).

Table S1. Comparison of anthropometric measurements and HGS with fat distribution indices and BIA-derived parameters.

Variable	HGS †		BMI		AC		CC	
	**≤Cutoff point** *n* = 33	**>Cutoff point** *n* = 70	**<23 kg/m²** *n* = 28	**≥23 kg/m²** *n* = 75	**≤22 cm** *n* = 8	**>22 cm** *n* = 95	**≤31cm** *n* = 12	**>31 cm** *n* = 91
	**M ± SD (95% CI)**	**M ± SD (95% CI)**	**M ±SD (95% CI)**	**M ± SD (95% CI)**	**M ± SD (95% CI)**	**M ± SD (95% CI)**	**M ± SD (95% CI)**	**M ± SD (95% CI)**
	**Cohen's *d***		**Cohen's *d***		**Cohen's *d***		**Cohen's *d***	
BAI	29.6 ± 5.8 * (27.6:31.7)	27.0 ± 6.4 * (25.5:28.5)	22.7 ± 4.2 *** (21.0:24.3)	29.7 ± 5.9 *** (28.4:31.1)	22.8 ± 4.9 * (18.8:26.9)	28.3 ± 6.2 * (27.0:29.6)	28.2 ± 6.0 (24.4:32.0)	27.8 ± 6.4 (26.5:29.1)
	d = 0.42		*d* = −1.29		*d* = −0.89		*d* = 0.06	
VAI	4.04 ± 3.24 (2.83:5.25)	3.79 ± 3.75 (2.88:4.69)	2.05 ± 1.41 ** (1.49:2.60)	4.55 ± 3.92 ** (3.63:5.48)	2.23 ± 2.01 (0.55:3.91)	4.01 ± 3.67 (3.24:4.78)	2.87 ± 2.45 (1.22:4.51)	3.99 ± 3.70 (3.20:4.78)
	d = 0.07		*d* = −0.73		*d* = −0.50		*d* = −0.31	
Post-dialysis body weight [kg]	70.9 ± 17.2 (64.8:77.0)	77.4 ± 21.3 (72.3:82.4)	57.8 ± 12.3 *** (53.0:62.6)	81.8 ± 18.7 *** (77.5:86.1)	48.6 ± 9.3 *** (40.7:56.4)	77.6 ± 19.3 *** (73.6:81.5)	59.5 ± 23.6 ** (44.5:74.1)	77.4 ± 18.9 ** (73.5:81.3)
	d = −0.32		*d* = −1.40		*d* = −1.55		*d* = −0.92	
Post-dialysis SMM [kg]	23.5 ± 5.5 *** (21.6:25.5)	29.1 ± 7.1 *** (27.4:30.8)	25.9 ± 8.1 (22.8:29.1)	27.8 ± 6.7 (26.3:29.3)	19.7 ± 4.8 ** (15.7:23.6)	27.9 ± 6.9 ** (26.5:29.4)	21.3 ± 9.8 ** (15.0:27.5)	28.1 ± 6.3 ** (26.8:29.4)
	d = −0.83		*d* = −0.26		*d* = −1.21		*d* = −1.00	
Post-dialysis lean body mass [kg]	44.1 ± 9.3 *** (40.8:47.4)	53.6 ± 11.4 *** (50.9:56.3)	48.8 ± 13.0 (43.7:53.8)	51.2 ± 11.1 (48.7:53.8)	38.1 ± 8.6 ** (31.0:45.3)	51.6 ± 11.3 ** (49.3:53.9)	40.7 ± 15.7 ** (30.8:50.7)	51.9 ± 10.4 ** (49.7:54.0)
	d = −0.88		*d* = −0.21		*d* = −1.22		*d* = −1.00	
Post-dialysis MBF [kg]	26.8 ± 12.1 (22.5:31.1)	24.1 ± 15.8 (20.3:27.9)	10.0 ± 4.7 *** (8.1:11.8)	30.5 ± 13.2 *** (27.5:33.6)	10.4 ± 4.7 ** (6.5:14.4)	26.2 ± 14.7 ** (23.2:29.2)	18.8 ± 10.6 (12.1:25.5)	25.8 ± 15.1 (22.6:28.9)
	d = 0.18		*d* = −1.78		*d* = −1.11		*d* = −0.48	
Post-dialysis fat tissue content (%)	36.5 ± 10.6 ** (32.7:40.2)	29.1 ± 13.0 ** (26.0:32.2)	17.6 ± 8.5 *** (14.3:20.9)	36.6 ± 9.8 *** (34.4:38.9)	21.8 ± 8.9 * (14.3:29.3)	32.3 ± 12.7 * (29.7:34.9)	30.4 ± 11.5 (23.2:37.7)	31.6 ± 12.9 (28.9:34.3)
	d = 0.60		*d* = −2.00		*d* = −0.84		*d* = −0.09	
Post-dialysis extracellular water index	0.40 ± 0.01 ** (0.39:0.40)	0.39 ± 0.02 ** (0.38:0.39)	0.39 ± 0.02 (0.38:0.40)	0.39 ± 0.01 (0.39:0.39)	0.40 ± 0.02 * (0.39:0.42)	0.39 ± 0.02 * (0.39:0.39)	0.40 ± 0.02 (0.38:0.41)	0.39 ± 0.02 (0.39:0.39)
	d = 0.57		*d* = 0.00		*d* = 0.50		*d* = 0.50	
Post-dialysis extracellular water [L]	12.9 ± 2.8 ** (11.9:13.9)	15.1 ± 3.3 ** (14.3:15.9)	13.7 ± 3.6 (12.3:15.1)	14.7 ± 3.1 (14.0:15.4)	11.4 ± 2.9 ** (9.0:13.8)	14.7 ± 3.2 ** (14.0:15.3)	11.6 ± 4.2 ** (9.0:14.2)	14.8 ± 3.0 ** (14.2:15.4)
	d = −0.70		*d* = −0.30		*d* = −1.03		*d* = −1.02	
Post-dialysis intracellular water [L]	19.6 ± 4.2 *** (18.1:21.1)	23.9 ± 5.4 *** (22.6:25.2)	21.4 ± 6.2 (19.0:23.8)	22.9 ± 5.1 (21.8:24.1)	16.6 ± 3.6 ** (13.6:19.7)	23.0 ± 5.3 ** (21.9:24.1)	17.9 ± 7.5 ** (13.1:22.6)	23.1 ± 4.9 ** (22.1:24.2)
	d = −0.85		*d* = −0.27		*d* = −1.23		*d* = −1.02	
Post-dialysis ECW/ICW ratio	0.66 ± 0.04 ** (0.65:0.68)	0.64 ± 0.04 ** (0.63:0.65)	0.65 ± 0.06 (0.62:0.67)	0.64 ± 0.04 (0.63:0.65)	0.68 ± 0.05 * (0.64:0.72)	0.64 ± 0.04 * (0.63:0.65)	0.66 ± 0.06 (0.62:0.69)	0.64 ± 0.04 (0.63:0.65)
	d = 0.50		*d* = 0.22		*d* = 0.98		*d* = 0.47	
Post-dialysis PA [°]	4.90 ± 1.15 *** (4.50:5.31)	6.00 ± 1.34 *** (5.68:6.32)	5.36 ± 1.72 (4.70:6.03)	5.75 ± 1.22 (5.47:6.03)	4.24 ± 1.08 ** (3.33:5.14)	5.76 ± 1.34 ** (5.49:6.04)	5.13 ± 2.18 (3.74:6.51)	5.71 ± 1.23 (5.46:5.97)
	d = −0.86		*d* = −0.28		*d* = −1.15		*d* = −0.42	

* $p < 0.05$; ** $p < 0.01$; *** $p < 0.001$. † HGS cutoff points for low muscle strength dependent on gender and BMI: women—BMI ≤ 23: ≤17 kg, BMI 23.1–26: ≤17.3 kg, BMI 26.1–29: ≤18 kg, BMI > 29: ≤21 kg; men—BMI ≤ 24: ≤29 kg, BMI 24.1–28: ≤30 kg, BMI > 28: ≤32 kg. Abbreviations: BIA—bioelectrical impedance analysis; *p*—probability testing; *n*—number of participants; M—arithmetic mean; SD—standard deviation; CI—confidence interval; *d*—Cohen's *d* effect size; HGS—handgrip strength; BMI—body mass index; AC—arm circumference; CC—calf circumference; BAI—body adiposity index; VAI—visceral adiposity index; SMM—skeletal muscle mass; MBF—mass of body fat (body fat mass); ECW/ICW—extracellular to intracellular water (extracellular water/intracellular water); PA—phase angle.

In the present study, patients classified according to the BMI < 23 kg/m² criterion exhibited lower values of BAI and VAI, as well as lower fat mass and fat tissue percentage, as determined through body composition analysis using bioelectrical impedance. A BMI below 23 kg/m² was associated with very large effect sizes (Cohen's *d*) for fat mass, fat tissue content, and adiposity indices (BAI and VAI), indicating substantial differences

between groups in fat-related parameters. In contrast, effect sizes for muscle and lean body mass were small, highlighting the limited diagnostic value of BMI for detecting muscle loss in dialysis patients. These findings emphasize the need to complement BMI with additional body composition measures. In previous studies, few reports addressed the use of BAI in patients with CKD or in older individuals, which may indicate that this index is a new, easily calculable, and useful tool that could be valuable in assessing body fat content and indicating overall adiposity.

Patients classified based on arm circumference (AC) below the cutoff point (AC ≤ 22 cm), compared to patients with AC > 22 cm, had significantly lower values of the following parameters: the BAI (22.82 ± 4.86 vs. 28.27 ± 6.24; $p = 0.018$); post-dialysis SMM (19.68 ± 4.75 kg vs. 27.93 ± 6.92 kg; $p = 0.001$); post-dialysis lean body mass (38.11 ± 8.57 kg vs. 51.61 ± 11.26 kg; $p = 0.001$); post-dialysis fat mass (10.44 ± 4.72 kg vs. 26.16 ± 14.65 kg; $p = 0.003$); post-dialysis fat tissue content (21.81 ± 8.93% vs. 32.27 ± 12.70%; $p = 0.025$); post-dialysis ICW (16.63 ± 3.63 L vs. 23.02 ± 5.30 L; $p = 0.001$); post-dialysis ECW (11.39 ± 2.89 L vs. 14.67 ± 3.19 L; $p = 0.006$); and post-dialysis PA (4.24 ± 1.08° vs. 5.76 ± 1.34°; $p = 0.002$). They also had significantly higher values of the post-dialysis ECW index (0.40 ± 0.02 vs. 0.39 ± 0.02; $p = 0.013$) and ECW/ICW ratio (0.68 ± 0.05 vs. 0.64 ± 0.04; $p = 0.012$). Notable differences between groups with low and normal AC were observed across most nutritional indicators. Very large effect sizes were found for post-dialysis body weight ($d = -1.55$), SMM ($d = -1.21$), lean body mass ($d = -1.22$), and ICW ($d = -1.23$), suggesting substantial muscle and fluid deficits in patients with low AC. Body fat mass ($d = -1.11$) and fat tissue content ($d = -0.84$) also showed large effects. These findings support AC as a reliable marker of global malnutrition involving both muscle and fat components (Table S1).

Patients classified based on calf circumference (CC) below the cutoff point (CC ≤ 31 cm), compared to patients with CC > 31 cm, had significantly lower values for the following parameters: the post-dialysis body weight (59.49 ± 23.64 kg vs. 77.38 ± 18.89 kg; $p = 0.004$); post-dialysis SMM (21.28 ± 9.83 kg vs. 28.09 ± 6.34 kg; $p = 0.002$); post-dialysis lean body mass (40.70 ± 15.65 kg vs. 51.86 ± 10.42 kg; $p = 0.002$); post-dialysis ICW (17.85 ± 7.51 L vs. 23.14 ± 4.85 L; $p = 0.001$); and post-dialysis ECW (11.60 ± 4.16 L vs. 14.79 ± 2.97 L; $p = 0.001$). Patients with low CC exhibited large to very large differences in key nutritional parameters. Lean body mass ($d = -1.00$), SMM ($d = -0.92$), ICW ($d = -1.02$), and the ECW/ICW ratio ($d = 0.98$) were among the most affected. These findings indicated substantial muscle loss and altered hydration balance in patients with low CC. Moderate effects were found for body fat mass ($d = -0.48$) and PA ($d = -0.42$), while fat tissue content showed minimal variation ($d = -0.09$), suggesting that CC better reflected muscle-related rather than fat-related deficits (Table S1).

Limb circumferences (AC and CC) demonstrated large to very large effect sizes in relation to muscle mass, fat mass (for AC), and hydration parameters—ICW and ECW—thereby confirming their utility as practical screening tools. As reported in previous studies, AC and CC may serve as simple and effective indicators of nutritional status, particularly in settings where access to advanced assessment methods is limited. In the study by El Hassani et al., it was found that AC was associated with lower PA values [42]. According to Da et al., both AC and CC may be useful in evaluating nutritional status. In HD patients, metabolic disturbances are observed, along with tissue loss, including a loss of body protein, fat mass, lean body mass, cellular mass, and muscle tissue mass [9].

The supplementary analyses provided additional insights that reinforce the main findings. While classical indicators such as BMI demonstrated limited utility in identifying muscle wasting, functional and bioimpedance-based measures such as HGS, PA, and ECW/ICW appeared to offer superior discriminatory value and may complement SGA-based nutritional assessments, being associated with nutritional status (Table S1).

Subsequent analyses involved the construction of a logistic regression model for the binary BMI variable, with the modeled class being BMI < 23 kg/m², based on the variables BAI and VAI (Table S2). In this analysis, receiver operating characteristic (ROC) curves were also generated for the BAI and VAI variables, with cutpoints determined using Youden's index (Figure S1).

Table S2. The logistic regression for the BMI variable, where the modeled variable was BMI < 23 kg/m² based on the BAI and VAI variables.

Variable	Result	Standard Error	Wald Test	p	OR	CI OR −95%	CI OR 95%
BAI	−0.328	0.075	18.968	<0.001	0.721	0.622	0.835
VAI	−0.456	0.156	8.504	0.004	0.634	0.467	0.861

$p < 0.05$. Abbreviation: OR—odds ratio.

An increase of one unit in the BAI variable reduced the odds of BMI < 23 kg/m² by 27.9%. The analysis of model goodness of fit, using the Hosmer–Lemeshow test (value = 8.32, p = 0.400) and the area under the receiver operating characteristic curve (AUC) value of 0.85, indicated that there was no evidence to suggest poor model fit. This was further supported by the ROC curve interpretation, with the cutpoint marked at 26.536 (Figure S1).

An increase of one unit in the VAI variable reduced the odds of BMI < 23 kg/m² by 36.6%. The analysis of model goodness of fit, using the Hosmer–Lemeshow test (value = 4.74, p = 0.790) and the AUC (0.74), indicated that there was no evidence to suggest poor model fit. This was further confirmed by the interpretation of the ROC curve, where the cutpoint was marked at 2.68 (Figure S1).

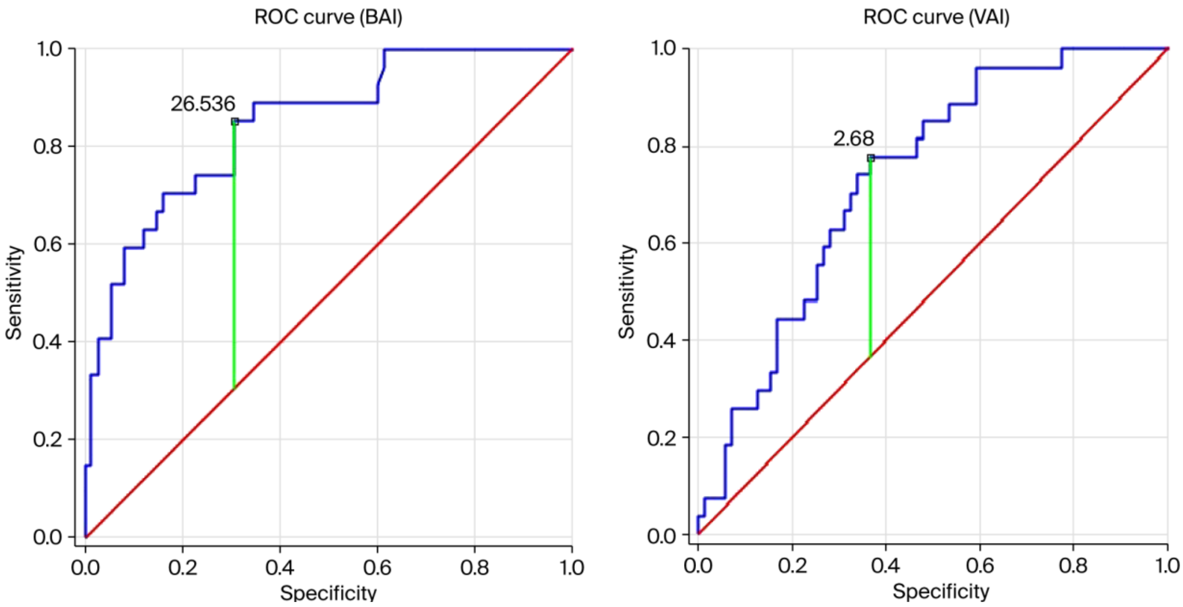

Figure S1. The receiver operating characteristic (ROC) curve for the modeled probability of BMI < 23 kg/m² based on the BAI and VAI.

The analysis of the results presented in Table S3 provided the basis for concluding that significantly higher proportions of individuals with an AC > 22 cm also had PA values > 5°, compared to those with a PA ≤ 5°. It was also observed that a greater proportion of individuals with normal fat tissue content—defined by waist-to-hip ratio (WHR) value (women: WHR < 0.8; men: WHR < 1)—were classified as having a BMI index < 23 kg/m². Furthermore, 100% of individuals with post-dialysis fat tissue content < 10% also had a BMI index < 23 kg/m².

Table S3. The observed frequencies for the associations of analyzed variables.

**Variable**	**Post-dialysis PA**		**χ^2; df; *p*-value; τ_b/φ_c; Cohen's κ**
**HGS** †	PA ≤ 5°, *n* (%)	PA > 5°, *n* (%)	χ^2_{Yates} = 12.87; df = 1; *p* < 0.001; τ_b = 0.37; κ = 0.376
≤Cutoff point, *n* (%)	19 (57.6%)	14 (20.0%)	
>Cutoff point, *n* (%)	14 (42.4%)	56 (80.0%)	
**AC**	PA ≤ 5°, *n* (%)	PA > 5°, *n* (%)	χ^2_{Yates} = 9.65; df = 1; *p* = 0.019; τ_b = 0.35; κ = 0.247
≤22 cm, *n* (%)	7 (21.2%)	1 (1.4%)	
>22 cm, *n* (%)	26 (78.8%)	69 (98.6%)	
**SGA**	PA ≤ 5°, *n* (%)	PA > 5°, *n* (%)	χ^2_{NW} = 31.37; df = 2; *p* < 0.001; φ_c = 0.55
Severely malnourished (SGA-C), *n* (%)	7 (21.2%)	0 (0.0%)	
Mild to moderately malnourished (SGA-B), *n* (%)	20 (60.6%)	25 (35.7%)	
Normal/well-nourished (SGA-A), *n* (%)	6 (18.2%)	45 (64.3%)	
**BMI**	**WHR** ‡		χ^2_{Yates} = 23.12; df = 1; *p* < 0.001; τ_b = 0.50; κ = 0.498
	Normal body fat content, *n* (%)	Visceral obesity, *n* (%)	
<23 kg/m², *n* (%)	17 (65.4%)	11 (14.3%)	
≥23 kg/m², *n* (%)	9 (34.6%)	66 (85.7%)	
**BMI**	**Post-dialysis body fat tissue content**		χ^2_{Yates} = 13.82; df = 1; *p* = 0.025; τ_b = 0.41; κ = 0.284
	Fat content < 10%, *n* (%)	Fat content ≥ 10%, *n* (%)	
<23 kg/m², *n* (%)	6 (100.0%)	22 (22.7%)	
≥23 kg/m², *n* (%)	0 (0.0%)	75 (77.3%)	

† HGS cutoff points for low muscle strength dependent on gender and BMI: women—BMI ≤ 23: ≤17 kg, BMI 23.1–26: ≤17.3 kg, BMI 26.1–29: ≤18 kg, BMI > 29: ≤21 kg; men—BMI ≤ 24: ≤29 kg, BMI 24.1–28: ≤30 kg, BMI > 28: ≤32 kg. ‡ WHR < 0.8 (women), WHR < 1 (men)—visceral fat tissue within normal range; WHR ≥ 0.8 (women), WHR ≥ 1 (men)—visceral obesity. Abbreviations: χ^2—chi-square test; τ_b—Kendall's tau-b coefficient; φ_c—Cramér's V; κ—Cohen's kappa coefficient; SGA—Subjective Global Assessment; WHR—waist-to-hip ratio.

Additionally, stratification by gender of patients participating in the study for the studied sociodemographic and clinical characteristics was performed, with results presented in Table S4.

Table S4. Sociodemographic and clinical characteristics of patients participating in the study (*n* = 103) in relation to gender.

Variable	Male<br>*n* = 64 (62.1%)<br>M ± SD (95% CI)<br>Me (IQR)	Female<br>*n* = 39 (37.9%)<br>M ± SD (95% CI)<br>Me (IQR)
Age [years] *	60 ± 15 (57:64)<br>63 (51–70)	63 ± 13 (59:68)<br>66 (51–74)
Education, n (%)		
Primary education	4 (6.3%)	7 (17.9%)
Vocational education	23 (35.9%)	9 (23.1%)
Secondary education	20 (31.3%)	17 (43.6%)
Higher education	17 (26.6%)	6 (15.4%)
Place of residence, n (%)		
Village	7 (10.9%)	5 (12.8%)
Small town	4 (6.3%)	3 (7.7%)
Medium-sized town	7 (10.9%)	6 (15.4%)
Large city	46 (71.9%)	25 (64.1%)
Marital status, n (%)		
Single	11 (17.2%)	6 (15.4%)
Married	41 (64.1%)	18 (46.2%)
Divorced	8 (12.5%)	1 (2.6%)
Widow/Widower	4 (6.3%)	14 (35.9%)
Social status, n (%)		
Unemployed	4 (6.3%)	2 (5.1%)
Employee	11 (17.2%)	5 (12.8%)
Retiree/Pensioner	49 (76.6%)	32 (82.1%)
Vascular access, n (%)		
Natural arteriovenous fistula	44 (68.8%)	26 (66.7%)
Permanent catheter	20 (31.3%)	13 (33.3%)
Presence of residual diuresis, n (%)	50 (78.1%)	32 (82.1%)
Hospitalization within the last year, n (%)	42 (65.6%)	25 (64.1%)
Cause of hospitalization, n (%) **		
COVID-19	5 (11.9%)	2 (8.0%)
Vascular access issues	27 (64.3%)	11 (44.0%)
Cardiovascular diseases	7 (16.7%)	5 (20.0%)
Others	15 (35.7%)	11 (44.0%)
Cause of CKD/comorbidities, n (%) **		
Diabetic kidney disease	18 (28.1%)	7 (17.9%)
Hypertensive nephropathy	34 (53.1%)	18 (46.2%)
Chronic glomerulonephritis	13 (20.3%)	12 (30.8%)
Others	52 (81.3%)	24 (61.5%)
	**M ± SD (95% CI)**<br>**Me (IQR)**	**M ± SD (95% CI)**<br>**Me (IQR)**
Dialysis vintage [months] *	68 ± 71 (50:85)<br>43 (16–82)	61 ± 67 (39:82)<br>39 (14–83)
Residual diuresis [mL] *	617.2 ± 623.9 (461.3:773.0)<br>450.0 (100.0–1000.0)	615.4 ± 612.1 (417.0:813.8)<br>500.0 (100.0–1000.0)

UFV [L]	2.3 ± 1.1 (2.0:2.6)<br>2.5 (1.3–3.2)	1.9 ± 1.0 (1.6:2.3)<br>1.9 (1.3–2.5)
Ideal body weight [kg]	72.2 ± 8.4 (70.0:74.3)<br>71.7 (67.5–78.1)	57.2 ± 6.5 (55.1:59.2)<br>57.3 (52.8–60.6)
Height [cm]	172.1 ± 8.1 (170.1:174.1)<br>171.7 (167.5–178.4)	157.5 ± 6.5 (155.4:159.6)<br>158.0 (152.8–161.0)
BMI [kg/m²] *	27.2 ± 6.5 (25.6:28.8)<br>26.0 (22.9–29.4)	26.9 ± 6.9 (24.7:29.2)<br>26.7 (21.1–33.6)
% Overweight *	16.5 ± 22.8 (10.8:22.2)<br>7.8 (0.0–22.3)	22.7 ± 25.0 (14.6:30.8)<br>17.3 (0.0–43.0)
BAI *	25.8 ± 6.1 (24.3:27.3)<br>25.7 (21.7–28.1)	31.2 ± 5.2 (29.5:32.8)<br>30.4 (26.5–35.7)
VAI *	4.20 ± 4.18 (3.13:5.28)<br>2.71 (1.35–4.75)	3.30 ± 2.25 (2.55:4.05)<br>3.08 (1.45–4.15)
WHR	1.01 ± 0.10 (0.98:1.03)<br>1.01 (0.93–1.08)	0.95 ± 0.12 (0.91:0.99)<br>0.92 (0.87–1.04)
Waist circumference [cm] *	99.2 ± 17.4 (94.9:103.5)<br>98.5 (85.8–110.3)	92.6 ± 18.2 (86.7:98.5)<br>87.0 (79.5–110.0)
HC [cm] *	98.2 ± 13.1 (94.9:101.5)<br>96.0 (91.5–103.0)	97.0 ± 10.0 (93.7:100.2)<br>97.0 (90.0–102.0)
AC [cm] *	29.2 ± 4.0 (28.2:30.3)<br>29.0 (26.8–31.3)	28.4 ± 5.0 (26.8:30.0)<br>29.0 (24.0–31.0)
CC [cm] *	36.7 ± 4.2 (35.6:37.8)<br>36.0 (35.0–38.8)	34.4 ± 4.7 (32.9:36.0)<br>35.0 (32.0–37.0)
Wrist circumference [cm] *	19.0 ± 1.5 (18.6:19.4)<br>19.0 (18.0–20.0)	17.6 ± 4.8 (16.1:19.2)<br>16.5 (16.0–18.0)
HGS [kg] *	34.4 ± 11.1 (31.6:37.2)<br>34.8 (26.3–41.4)	19.1 ± 5.0 (17.5:20.7)<br>19.4 (15.7–21.6)
URR (%) *	72.5 ± 5.4 (71.1:73.8)<br>73.6 (69.0–76.3)	78.1 ± 5.5 (76.3:79.9)<br>79.0 (74.5–82.6)
Pre-dialysis urea concentration [mmol/L] *	20.2 ± 5.3 (18.9:21.5)<br>19.5 (17.4–22.3)	17.8 ± 5.8 (16.0:19.7)<br>17.2 (13.9–20.8)
Post-dialysis urea concentration [mmol/L] *	5.7 ± 2.2 (5.1:6.2)<br>5.1 (4.3–6.6)	4.0 ± 1.8 (3.4:4.5)<br>3.5 (2.6–5.2)
Post-dialysis body weight [kg] *	80.6 ± 20.1 (75.6:85.6)<br>77.1 (68.4–87.7)	66.6 ± 17.4 (61.0:72.3)<br>65.8 (52.6–81.0)
Post-dialysis protein [kg]	10.9 ± 1.9 (10.4:11.3)<br>10.9 (9.5–12.1)	7.9 ± 1.9 (7.3:8.5)<br>7.6 (7.0–8.4)
Post-dialysis SMM [kg]	30.7 ± 5.7 (29.3:32.1)<br>30.7 (26.7–34.5)	21.7 ± 5.6 (19.9:23.5)<br>21.0 (19.0–23.2)
Post-dialysis lean body mass [kg]	56.0 ± 9.3 (53.7:58.4)<br>55.5 (48.8–62.1)	41.6 ± 9.3 (38.6:44.6)<br>39.8 (36.6–44.3)
Post-dialysis MBF [kg] *	24.5 ± 16.0 (20.5:28.5)<br>21.9 (13.5–30.9)	25.7 ± 12.5 (21.6:29.7)<br>26.2 (13.0–37.9)
Post-dialysis fat tissue content (%)	28.5 ± 12.1 (25.5:31.5)<br>30.3 (20.0–37.1)	36.3 ± 12.3 (32.3:40.3)<br>38.2 (27.3–45.1)
Post-dialysis extracellular water [L]	16.0 ± 2.6 (15.4:16.7)<br>15.9 (14.3–17.5)	11.8 ± 2.5 (11.0:12.6)<br>11.4 (10.6–12.5)
Post-dialysis intracellular water [L]	25.2 ± 4.3 (24.1:26.2)<br>25.1 (22.1–28.0)	18.2 ± 4.3 (16.8:19.6)<br>17.6 (16.1–19.3)
Post-dialysis extracellular water index	0.390 ± 0.015 (0.386:0.393)<br>0.389 (0.378–0.402)	0.393 ± 0.017 (0.388:0.399)<br>0.394 (0.385–0.404)
Post-dialysis ECW/ICW ratio	0.640 ± 0.041 (0.630:0.650)<br>0.638 (0.610–0.669)	0.649 ± 0.047 (0.634:0.665)<br>0.650 (0.623–0.676)

Post-dialysis PA [°]	5.88 ± 1.24 (5.57:6.18)<br>5.85 (4.90–6.70)	5.27 ± 1.52 (4.78:5.76)<br>5.20 (4.50–5.80)
Creatinine [mg/dL] *	9.74 ± 2.38 (9.15:10.34)<br>9.56 (7.86–11.70)	7.40 ± 2.19 (6.67:8.13)<br>7.72 (5.96–9.00)
Albumin [g/L] *	39.5 ± 3.3 (38.6:40.3)<br>40.0 (37.0–41.9)	36.8 ± 4.5 (35.4:38.3)<br>37.9 (35.3–39.7)
Total protein [g/L]	65.5 ± 4.8 (64.3:66.8)<br>65.8 (63.1–67.8)	65.8 ± 4.2 (64.4:67.2)<br>65.8 (63.1–68.6)
Transferrin [g/L] *	1.77 ± 0.35 (1.69:1.86)<br>1.76 (1.54–1.93)	1.67 ± 0.29 (1.58:1.77)<br>1.72 (1.46–1.80)
Total cholesterol [mmol/L] *	4.10 ± 1.13 (3.81:4.39)<br>3.97 (3.45–4.49)	4.69 ± 1.33 (4.24:5.13)<br>4.31 (3.52–5.57)
HDL [mmol/L] *	1.05 ± 0.43 (0.94:1.16)<br>0.94 (0.75–1.27)	1.21 ± 0.51 (1.04:1.38)<br>1.05 (0.88–1.37)
LDL [mmol/L] *	2.42 ± 0.88 (2.19:2.64)<br>2.28 (1.85–3.03)	2.90 ± 1.21 (2.50:3.30)<br>2.56 (1.97–4.04)
TG [mmol/L] *	1.63 ± 1.20 (1.32:1.94)<br>1.26 (0.77–1.96)	1.68 ± 0.80 (1.41:1.94)<br>1.56 (1.16–2.05)
hs-CRP [mg/L] *	9.7 ± 16.0 (5.6:13.8)<br>4.1 (1.4–11.7)	7.5 ± 11.1 (3.9:11.1)<br>4.3 (1.4–6.9)
nPCR [g/kg/day]	1.05 ± 0.22 (0.99:1.10)<br>1.04 (0.92–1.19)	0.98 ± 0.23 (0.91:1.06)<br>0.99 (0.82–1.11)

Note: Results are the M ± SD (and 95% CI for the mean) and Me (25th–75th percentiles) for continuous variables or numbers (and percentages) for categorical variables. * Non-normally distributed data (Shapiro–Wilk test, $p < 0.05$). ** Some patients experienced more than one event. Abbreviations: Me—median; IQR—interquartile range; COVID-19—coronavirus disease 2019; CKD—chronic kidney disease; UFV—ultrafiltration volume; HC—hip circumference; URR—urea reduction ratio; HDL—high-density lipoprotein; LDL—low-density lipoprotein; TG—triglycerides; hs-CRP—high-sensitivity C-reactive protein; nPCR—normalized protein catabolic rate.
